# The Curse of Black Sigatoka: A Backward Bifurcation Perspective

**DOI:** 10.1007/s11538-026-01769-2

**Published:** 2026-10-11

**Authors:** Bernard Asamoah Afful, Luis F. Gordillo

**Affiliations:** Department of Mathematics and Statistics, Utah State University, Logan, UT USA

**Keywords:** Black Sigatoka disease, Black leaf streak disease, Banana fungal airborne disease, Basic reproduction number, Stochastic simulation algorithm

## Abstract

Black Sigatoka disease (BSD), also known as black leaf streak disease, is an airborne fungal infection caused by *Pseudocercospora fijiensis* that severely impacts global banana and plantain production. Its persistence and resistance to eradication make it one of the most challenging plant diseases to manage. In this paper, we propose a deterministic pathogen-host model to describe BSD dynamics. Due to dual transmission pathways (ascospores and conidia) and mate limitation in sexual reproduction, the model exhibits a backward bifurcation: a stable endemic equilibrium coexists with the disease-free equilibrium for certain parameter values in which the basic reproduction number, $\mathcal {R}_0$, is less than 1. This phenomenon explains why control strategies that solely reduce $\mathcal {R}_0$ below one may fail. For the backward bifurcation regime, we perform sensitivity analysis of the endemic equilibrium using normalized forward sensitivity indices, Latin Hypercube Sampling, and Partial Rank Correlation Coefficients. Results indicate that effective control must extend beyond $\mathcal {R}_0$ reduction and prioritize (1) limiting production of new susceptible leaves during high-risk periods and (2) developing and deploying disease-resistant plant varieties. To incorporate transmission variability, we also formulate a stochastic version of the model using the Stochastic Simulation Algorithm (SSA). Extensive numerical simulations compare stochastic realizations with deterministic predictions and quantify variability in disease dynamics. To identify the principal drivers of persistence and variability, we analyze the endemic equilibrium using Sobol’s variance-based sensitivity method, which highlights the role of nonlinear parameter interactions in shaping variability.

## Introduction

Black Sigatoka disease (BSD), also known as black leaf streak disease (BLSD), is an airborne fungal disease caused by *Pseudocercospora fijiensis* (formerly *Mycosphaerella fijiensis*). It is endemic in most banana- and plantain-producing regions worldwide and ranks among the most severe threats to global banana production. In susceptible cultivars, the pathogen induces extensive leaf necrosis and premature defoliation before fruit bunches reach maturity, resulting in yield losses that frequently exceed 50% in the absence of control measures.

Despite intensive management efforts, BSD remains exceptionally difficult to eradicate or suppress effectively. Several factors contribute to this resilience: the pathogen produces abundant ascospores that facilitate long-distance wind dispersal and genetic recombination; resistance to multiple fungicide classes has emerged following repeated applications; and the fungus is well adapted to warm, humid tropical environments that promote short disease cycles and frequent reinfection. These characteristics make containment or complete eradication nearly impossible in most production systems. Reliance on frequent fungicide sprays achieves only partial suppression, at considerable economic and environmental cost.

A rare exception occurred in North Queensland, Australia. Following the detection of BSD in April 2001 in the Tully region of Australia, the country’s largest commercial banana-growing area, an intensive, multi-year eradication campaign was implemented. This program involved rigorous surveillance, destruction of infected host material where necessary, and strict quarantine measures. The effort successfully eliminated the pathogen from commercial plantations, representing perhaps the only documented instance of BSD eradication from a major production zone.

BSD spreads via two spore types, asexual conidia and sexual ascospores, which play complementary roles in the epidemic cycle. Conidia, produced in young to intermediate lesions, drive rapid local spread through rain splash, water runoff, or localized wind, sustaining polycyclic progression within plantations. Ascospores, maturing on necrotic tissue and forcibly discharged under wet conditions, enable broader geographic dispersal (often tens to hundreds of kilometers) and serve as primary inoculum for initiating epidemics in new areas or reinfecting plantations after seasonal lows or sanitation efforts. The combination of these dispersal mechanisms creates a highly effective transmission system.

The use of a mathematical model helps study population and disease transmission dynamics, understand the complex system that includes humans, animals, and plants, and investigate intervention measures and improve health control strategies (Tresna et al. 2023). Mathematical modeling of plant diseases has made significant strides over time (Cunniffe et al. 2015; Jeger et al. 2007; Tresna et al. 2023). Several authors have developed models that describe the transmission dynamics and control of plant diseases (Anggriani et al. 2018; Bokil et al. 2019; Tresna et al. 2023; Venturino et al. 2016). Regarding diseases affecting bananas and plantains, there is a limited number of models available in the literature. Recent progress has included the development of spatially explicit models that capture the heterogeneity of disease spread in agricultural landscapes (Abadie et al. 2024), time-delay models accounting for latency in transmission (Agouanet et al. 2021), and deterministic frameworks for analyzing threshold conditions and control strategies (Agouanet et al. 2024; Mapinda et al. 2025). Recent advances have also introduced machine learning techniques for the early detection and forecasting of plant disease outbreaks (Genet et al. 2025; Ugarte Fajardo et al. 2020, 2022; Wang et al. 2020). Meanwhile, other studies have explored alternative modeling paradigms to enhance prediction and intervention design (Elinisa et al. 2025; Freire et al. 2023; Henao-Ochoa et al. 2025; Olivares et al. 2022; Rehman et al. 2025; Said et al. 2021; Singh et al. 2024; Strobl and Mohan 2020; Sujatha et al. 2025; Thiagarajan et al. 2024; Uchôa et al. 2021; Yonow et al. 2019).

Despite these advances, several important biological mechanisms relevant to BSD remain insufficiently represented in existing mathematical models. In particular, previous deterministic models generally assume a single transmission pathway or linear pathogen reproduction and therefore cannot capture the interaction between local rain-splash dispersal by conidia and long-distance wind dispersal by ascospores. Furthermore, the heterothallic reproductive biology of *Pseudocercospora fijiensis*, which requires compatible mating types for sexual reproduction, has not been explicitly incorporated into compartmental epidemic models through mate-limitation or Allee-effect mechanisms. Consequently, previous studies do not explain how BSD may persist even when the basic reproduction number is below unity, nor do they investigate the stochastic persistence and eradication probabilities associated with these nonlinear transmission processes.

In this paper, we present a quantitative compartmental pathogen-host model using differential equations to describe BSD dynamics. The model incorporates both spore types and accounts for mate limitation in ascospore production (due to the nature of *Pseudocercospora fijiensis*, which requires compatible mating types). We derive the basic reproduction number, $\mathcal {R}_0$, and demonstrate that the model exhibits a backward bifurcation, by which a stable endemic equilibrium coexists with the disease-free equilibrium for $\mathcal {R}_0<1$. This bistability provides a theoretical explanation for the disease’s extraordinary resilience and the failure of control strategies that aim solely to reduce $\mathcal {R}_0$ below 1. We also formulate a stochastic model, implementing the Stochastic Simulation Algorithm (SSA) (Gillespie 1977), to investigate how intrinsic, individual randomness affects the overall variability of the disease dynamics.

The paper is structured as follows. In Sects. 2 and 5, we describe the deterministic and stochastic compartmental models, respectively. Section 3 derives the disease-free and endemic equilibria, computes $\mathcal {R}_0$, and analyzes backward bifurcation. In Sect. 4, we conduct sensitivity analysis of the endemic equilibrium using normalized forward sensitivity indices, Latin Hypercube Sampling, and Partial Rank Correlation Coefficients. Section 5 presents comprehensive numerical simulations that compare deterministic predictions with stochastic realizations, examine persistence/eradication thresholds under varying environmental and biological conditions, assess eradication probabilities at small population sizes, and use Sobol’s variance-based method for quantifying the contributions of individual parameters and their interactions to the variability of the endemic equilibrium. Finally, the conclusions are presented in Sect. 6.

## A deterministic Model

**Fig. 1:**
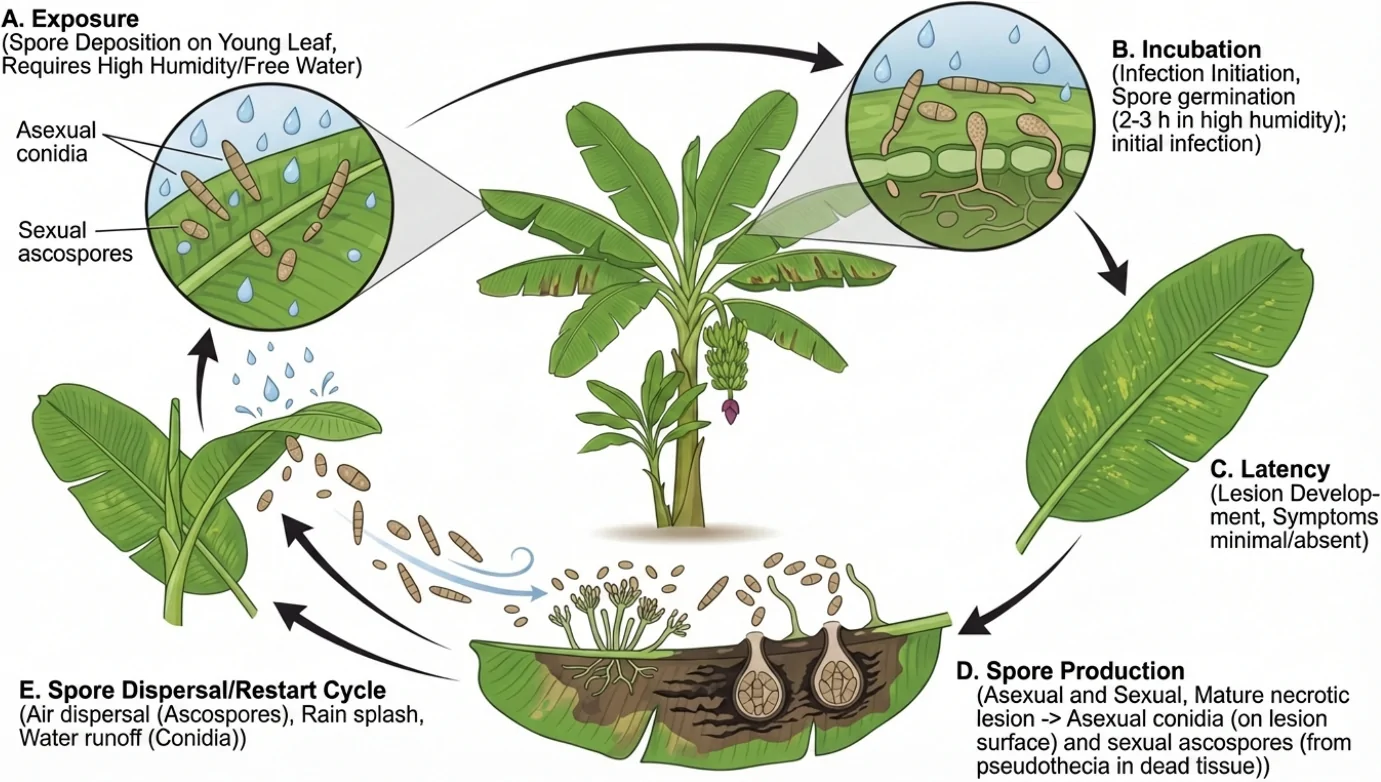
Life cycle of Black Sigatoka disease (BSD), caused by *Pseudocercospora fijiensis* on a banana plant. The diagram illustrates the progression from spore deposition on young leaves through infection, incubation, latency, asexual spore (conidia) production within developing lesions, and eventual sexual maturation on necrotic tissue, culminating in ascospore production in pseudothecia. Both conidia and ascospores serve as inoculum for new infections. Arrows indicate key transitions and spore dispersal pathways. (Adapted from Ravigné et al. (2017) using (figurelabs.ai)) (color figure online)

The life cycle of the causal agent of Black Sigatoka disease (BSD), *Pseudocercospora fijiensis* (formerly *Mycosphaerella fijiensis*), is illustrated in Fig. 1. The fungus completes its entire infection cycle on the banana plant, progressing through four distinct phases: (1) exposure, (2) incubation, (3) latency, and (4) spore production. As with many foliar fungal pathogens, spore germination and infection require high relative humidity or a wet leaf surface. Consequently, relative humidity and temperature strongly influence disease progression. In BSD, leaf susceptibility decreases with age, making young leaves the primary sites for spore deposition and germination. Following germination, lesions develop through exposure and latency phases before producing asexual conidia and, upon maturation of necrotic tissue, sexual ascospores. Spore germination typically occurs within 2–3 h under conditions of high humidity and free water availability during the exposure phase.

Let *N*(*t*) denote the total leaf biomass density at time *t*. This biomass is partitioned into four compartments: susceptible *H*(*t*), resistant *R*(*t*), exposed *E*(*t*), and infected *I*(*t*), that is, N(t)=H(t)+R(t)+E(t)+I(t). Healthy leaf biomass consists of susceptible and resistant biomass, H(t)+R(t). Susceptible leaves permit rapid spore germination and infection under favorable climatic conditions, whereas resistant leaves inhibit initial spore development, even in suitable environments; spores on resistant leaves may fail to germinate or do so slowly. New leaf biomass enters the system at a rate Λ. The fraction of susceptible leaves is denoted by κ (with 1-κ resistant). The natural decay rate of leaves is assumed constant and denoted by μ.

### The Transmission Rate

BSD is transmitted via two complementary spore types: asexual conidia and sexual ascospores. The pathogen exhibits a polycyclic life cycle, with multiple infection generations possible within a single growing season. Both spore types initiate infections on susceptible leaves, producing new lesions and perpetuating transmission.

Conidia, produced in young to intermediate lesions, are dispersed primarily over short distances by rain splash, water runoff, or localized wind. They drive rapid local epidemics and sustain polycyclic progression within plantations. Conidia germinate under high humidity and free water on leaf surfaces, penetrating stomata to form new lesions. Although fewer conidia are produced per lesion than ascospores, they are essential for building local epidemic intensity.

Ascospores mature on necrotic leaf tissue and are forcibly discharged under wet conditions. Smaller and more abundant per lesion, ascospores are primarily wind-dispersed, enabling long-distance movement (tens to hundreds of kilometers, though limited by UV sensitivity). They serve as the primary inoculum for initiating epidemics in new areas or reinfecting plantations after sanitation or seasonal declines, while also contributing to within-plantation spread and genetic recombination (e.g., facilitating fungicide resistance adaptation).

The densities of conidia and ascospores at time *t* are denoted by *A*(*t*) and *S*(*t*), respectively. The transmission rate β depends on relative humidity *h* and temperature *T*,

1 $$\begin{aligned} \beta (h,T)=\beta _0\overbrace{\left( \frac{h}{h+K_h}\right) }^{\text {Humidity effects}}\underbrace{\left( \exp {\left( -\frac{(T-\hat{T})^2}{2\sigma _T^2}\right) }\right) }_{\text {Temperature effects}}, \quad \beta _0,K_h,\hat{T},\sigma ^2_T>0. \end{aligned}$$

Here, $K_h$ denotes the half-saturation constant describing the humidity response of the transmission rate and should not be confused with the recruitment parameter κ, which represents the fraction of newly recruited susceptible leaves.

The rate of new infections is modeled as

2 $$\begin{aligned} \Gamma =\beta (h, T)\theta SH + \beta (h,T)\psi A H/N, \end{aligned}$$

where $\theta , \psi > 0$ and N=N(t) is the total leaf biomass. The first term employs mass-action incidence to represent long-distance, density-independent transmission by wind-dispersed ascospores, while the second term employs standard incidence to capture localized, frequency-dependent spread by rain-splash-dispersed conidia.

**Fig. 2:**
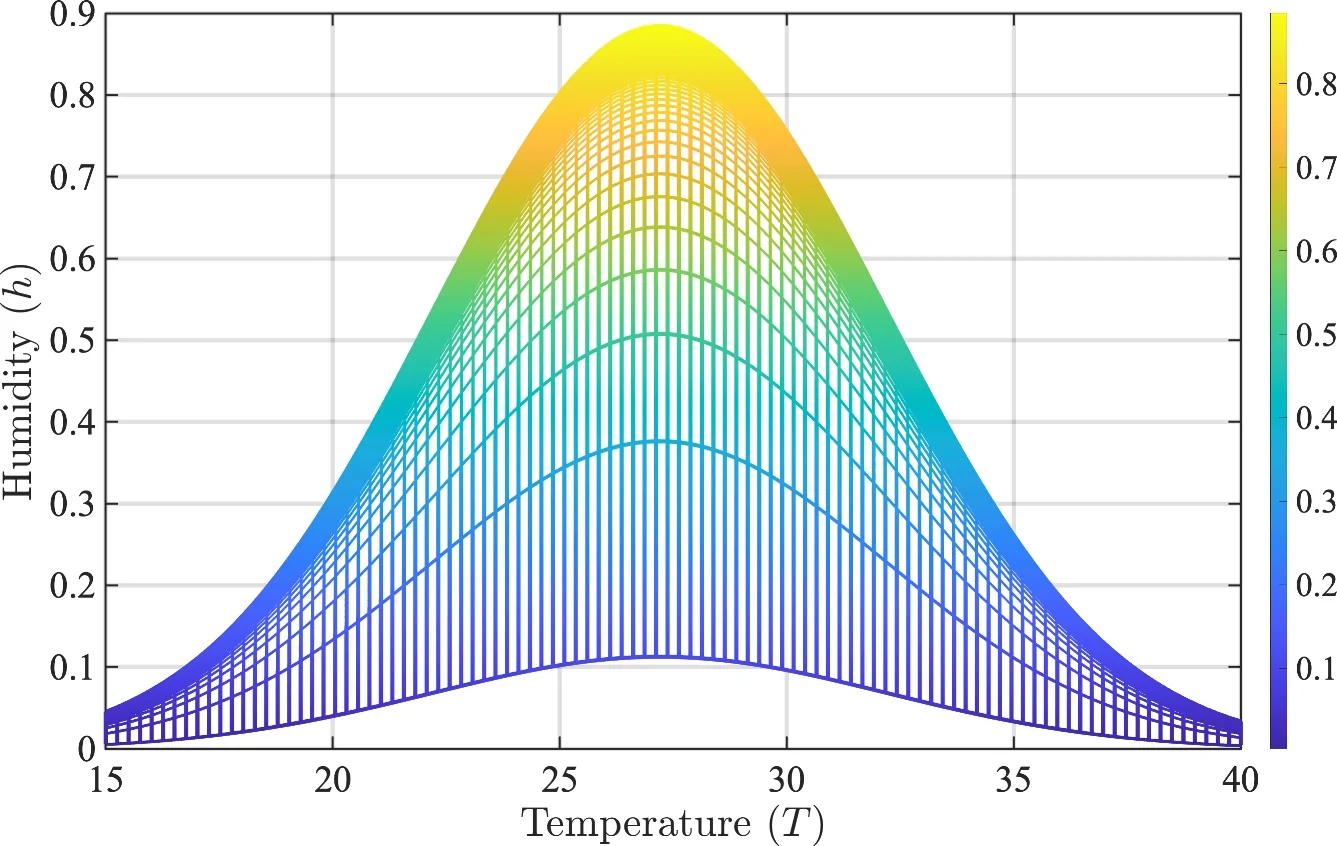
Climate-dependent transmission rate β(h,T) as a function of temperature and relative humidity. The color scale indicates the relative transmission rate, with warmer colors corresponding to higher transmission and cooler colors corresponding to lower transmission (color figure online)

The environmental dependence incorporates saturation at high humidity and a Gaussian peak around the optimal temperature $\hat{T}$ (see Fig. 2), providing a biologically realistic representation of transmission under varying climatic conditions (Hridoy 2025; Ramírez-Soto et al. 2023).

### Mate Limitation in Ascospores

*Pseudocercospora fijiensis* is heterothallic, requiring two compatible mating types (MAT1-1 and MAT1-2, typically in a balanced ratio in established populations) for successful sexual reproduction (Alby et al. 2009). Ascospores form in pseudothecia on necrotic tissue following fertilization of receptive hyphae by spermatia of the opposite mating type. This process requires physical proximity between compatible mycelia within or between lesions.

At high densities of infected leaf biomass, mating opportunities are abundant, allowing ascospore production to approach its maximum rate. At low densities, such as during early invasion, post-sanitation recovery, or in small or fragmented plantations, the encounter rate between compatible mating types declines sharply, introducing an Allee effect due to mate limitation, whereby reproductive success increases with population density until compatible mates become sufficiently abundant. Similar density-dependent mating mechanisms have been reported for several fungal pathogens and have been incorporated into mathematical models of fungal population dynamics and invasion biology (Lehtonen and Schwanz 2018; Hamelin et al. 2016; Suffert et al. 2019). The per-unit-biomass rate of successful mating, *g*(*I*), therefore decreases nonlinearly with declining density.

The Allee effect considered here arises from density-dependent mate limitation rather than from the relative abundance of mating types alone. At low pathogen densities, infected lesions are less likely to contain compatible mating types, reducing sexual reproduction and lowering the pathogen’s per-capita growth rate. As infected biomass increases, mating opportunities become more frequent until saturation is reached because of finite lesion capacity. From a biological perspective, this positive density dependence amplifies differences in subpopulation growth rates, allowing small pathogen populations to remain reproductively disadvantaged while larger populations sustain epidemic growth.

The rate of new ascospore production is thus αg(I(t))I(t), where α>0 and

3 $$\begin{aligned} g(I(t))=\frac{\lambda I(t)}{1+\lambda I(t)},\quad \lambda >0. \end{aligned}$$

**Fig. 3:**
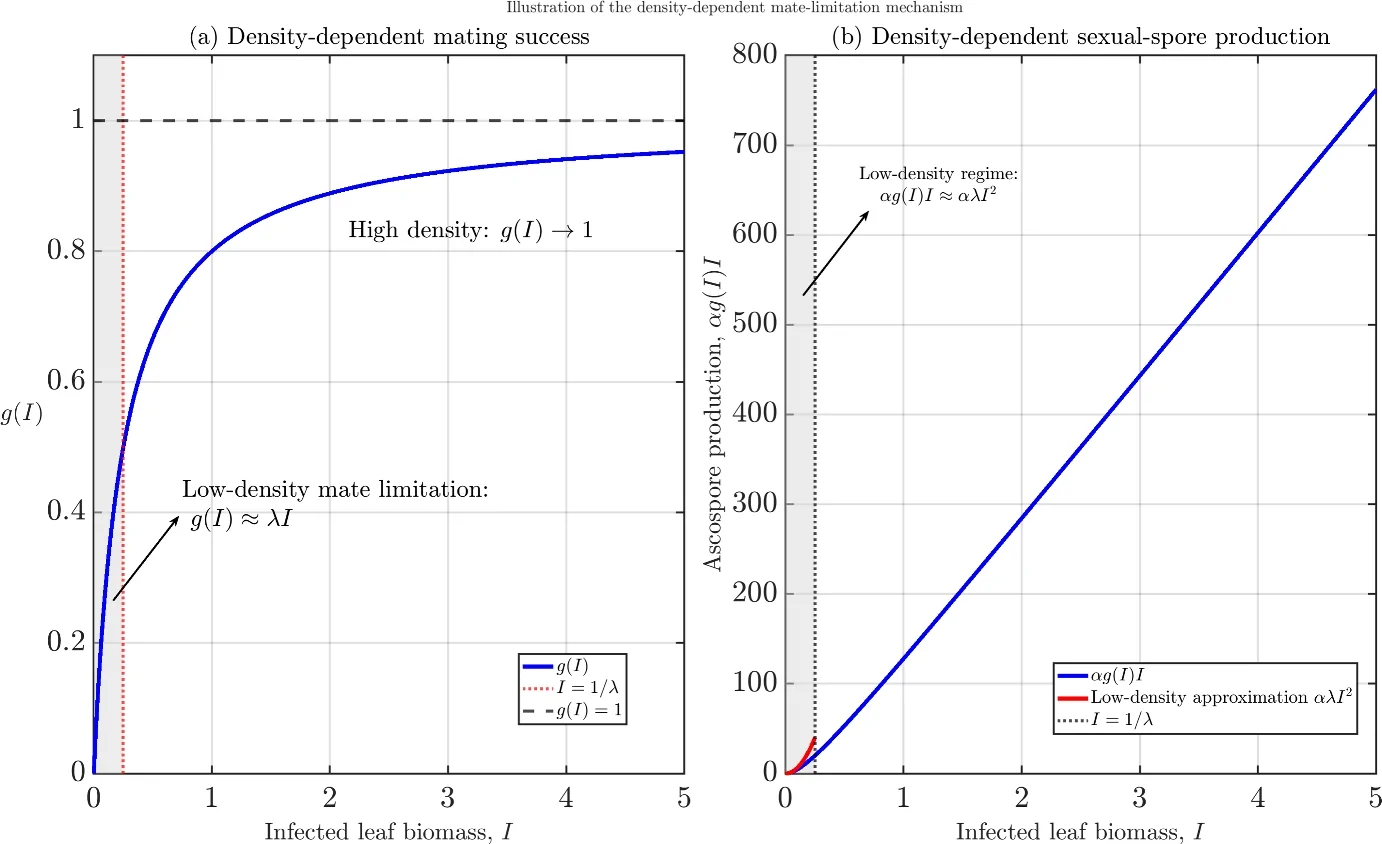
Illustration of the density-dependent mate-limitation mechanism incorporated into the Black Sigatoka disease model. **a** Mating success function *g*(*I*), showing reduced mating success at low infected leaf biomass and saturation at high biomass. **b** Corresponding ascospore production rate, αg(I)I, highlighting the low-density quadratic approximation $\alpha \lambda I^2$ resulting from mate limitation (color figure online)

When $I(t)\gg 1/\lambda$ (high density), g(I(t))≈1, yielding near-maximal production. When $I(t)\ll 1/\lambda$ (low density), $g(I(t))\approx \lambda I(t)$, so ascospore production scales quadratically as $\alpha \lambda I(t)^2$, reflecting the pairwise requirement for compatible mates. Figure 3 illustrates how mate limitation suppresses sexual reproduction when infected leaf biomass is low because compatible mating types are less likely to encounter one another. As infected biomass increases, mating success approaches its maximum, leading to increased ascospore production. This density-dependent reproductive mechanism introduces an Allee effect that contributes to pathogen persistence and the emergence of backward bifurcation in the model.

Although other monotone saturation functions could be used (Ravigné et al. 2017; Lehtonen and Schwanz 2018; Hamelin et al. 2016), the functional form (3) is mathematically simple, biologically interpretable, and has been widely employed to represent encounter-limited biological processes. The qualitative results presented in this work depend on the presence of density-dependent mate limitation rather than on the particular analytical expression adopted for *g*(*I*).

Conidia are produced at a constant rate η per unit infected biomass. Infections initially produce latent lesions unable to generate spores until maturity (rate γ). Cultural practices (such as pruning, sanitation, and removal of infected plant material) reduce infected leaf biomass and both asexual and sexual spore biomass at a constant per-unit biomass rate ρ. Consequently, the removal terms ρI, ρA, and ρS represent biomass-dependent losses resulting from disease management interventions.

### The Model

**Fig. 4:**
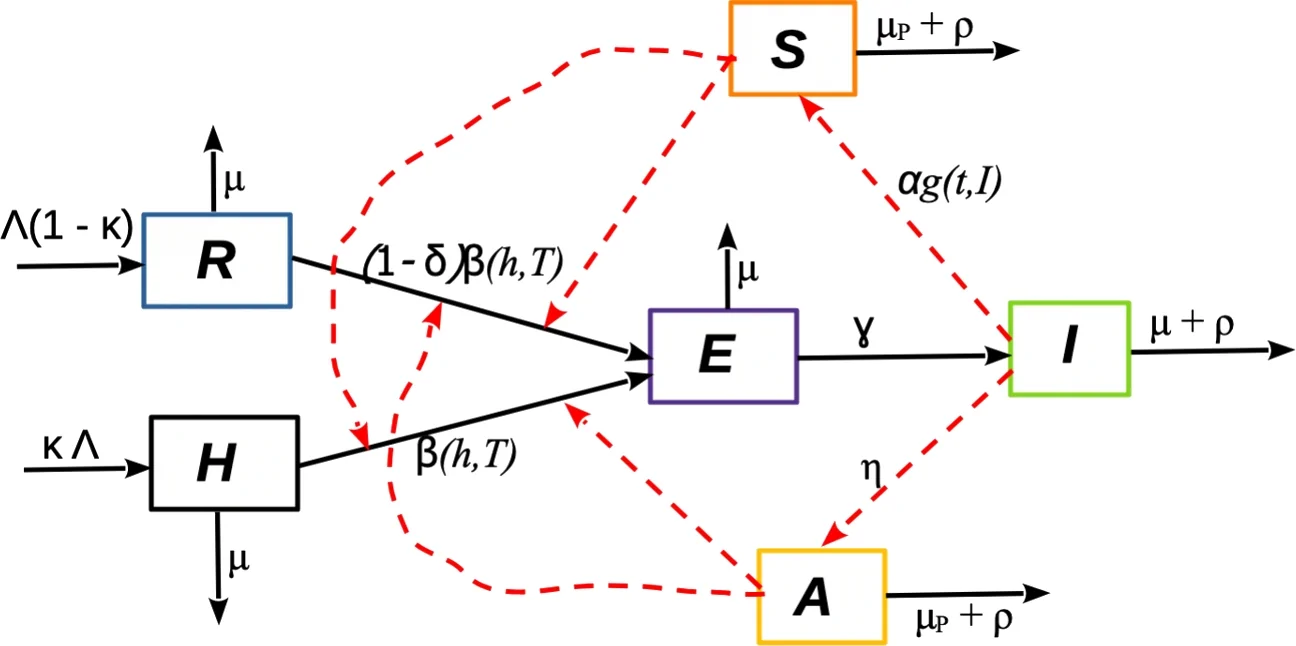
Schematic diagram showing the dynamics of the proposed Black Sigatoka Disease model. Solid arrows represent the flow of leaf biomass through the disease compartments; dashed arrows indicate the interaction between leaves and spores (color figure online)

Figure 4 presents a schematic of the compartmental dynamics. Combining the above assumptions yields the following system of differential equations

4 $$\begin{aligned} {{\textbf {Host population}}}&{\left\{ \begin{array}{ll} \dfrac{dH}{dt}=\kappa \Lambda - \beta (h,T)(\theta S+\psi \frac{A}{N})H - \mu H,\\ \dfrac{dR}{dt}=(1-\kappa )\Lambda - (1-\delta )\beta (h,T)(\theta S+\psi \frac{A}{N})R - \mu R,\\ \dfrac{dE}{dt}=\beta (h,T)(\theta S+\psi \frac{A}{N})(H+(1-\delta )R) - (\gamma +\mu )E,\\ \dfrac{dI}{dt}=\gamma E - (\mu +\rho )I. \end{array}\right. } \end{aligned}$$

5 $$\begin{aligned} {{\textbf {Pathogen population}}}&{\left\{ \begin{array}{ll} \dfrac{dA}{dt}=\eta I - (\mu _P+\rho )A,\\ \dfrac{dS}{dt}=\alpha g(I)I - (\mu _P+\rho )S. \end{array}\right. } \end{aligned}$$

The model accounts for resistance efficacy to the disease, δ, $0\le \delta \le 1$, which quantifies the ability of resistant leaves to suppress spore penetration and germination. These equations capture the essential biological and environmental drivers of BSD transmission while incorporating resistance, dual spore pathways, and density-dependent sexual reproduction. Parameter definitions, dimensions, and values are provided in Tables 1 and 2.

**Table 1:** The BSD model’s parameters and their respective descriptions and dimensions

	Description	Dimension
Variables		
*H*	Biomass of susceptible leaves	Leaves per ha
*R*	Biomass of resistant leaves	Leaves per ha
*E*	Biomass of exposed leaves	Leaves per ha
*I*	Biomass of infected leaves	Leaves per ha
*A*	Biomass of asexual spores	Spores per unit of leaf biomass
*S*	Biomass of sexual spores	Spores per unit of leaf biomass
Parameters		
Λ	Recruitment or replanting rate of healthy leaves	Leaves per day
κ	Fraction of susceptible recruited plants	Dimensionless
δ	Efficacy rate of leaf resistance	Dimensionless
$\beta _0$	Base transmission rate	$\textrm{day}^{-1}$
β(h,T)	Effective disease transmission rate	$\textrm{day}^{-1}$
γ	Latency period	$\textrm{day}^{-1}$
θ	Sexual spore dispersal rate	$\mathrm{(spores} \times \mathrm{day)}^{-1}$
ψ	Asexual spore dispersal rate	$\mathrm{(spores }\times \mathrm{day)}^{-1}$
η	Asexual spore production rate	Spores per leaf per day
α	Sexual spore production rate	Spores per leaf per day
μ	Natural decay of leaves	$\textrm{day}^{-1}$
$\mu _P$	Natural mortality of pathogen	$\textrm{day}^{-1}$
ρ	Removal rate due to cultural management	$\textrm{day}^{-1}$
	(pruning, sanitation, removal of infected tissue)	
λ	Maximum achievable mating rate	Mating per lesion
$\sigma _T$	Temperature tolerance	degrees Celsius (C)
*h*	Humidity level	Dimensionless
$K_h$	Half-saturation constant for humidity	Dimensionless
*T*	Temperature	degrees Celsius (C)
$\hat{T}$	Optimal temperature	degrees Celsius (C)

**Table 2:** Parameter values of the BSD model (4) with description in Table 1

Parameters	Values	References	Parameters	Values	References
Λ	20	Agouanet et al. (2024)	μ	0.01	Ravigné et al. (2017)
κ	0.5	Agouanet et al. (2024)	$\mu _P$	0.1	Assumed
δ	0.9	Agouanet et al. (2024)	ρ	0.5	Assumed
$\beta _0$	0.9	Assumed	λ	4	Agouanet et al. (2024)
γ	0.01	Ravigné et al. (2017)	$\sigma _T$	5	Assumed
η	20	Ravigné et al. (2017)	*h*	0.9	Uchôa et al. (2021)
α	50	Agouanet et al. (2024)	$\hat{T}$	27.2	Bebber (2019)
θ	48	Agouanet et al. (2024)	*T*	30.3	Bebber (2019)
ψ	20	Agouanet et al. (2024)	$K_h$	0.7	Yonow et al. (2019)

Parameters marked “Assumed" in Table 2 were selected to remain consistent with biologically plausible ranges reported in related fungal plant-pathogen modeling studies, particularly those addressing transmission, environmental forcing, and control processes in foliar diseases; the robustness of the model outcomes to these assumptions is subsequently assessed through local, global, and variance-based sensitivity analysis in Sects. 4 and 5.

## Basic Reproduction Number

### BSD-Free and BSD-Persistent Equilibria

The disease-free equilibrium corresponds to the absence of pathogen infection. Accordingly, the exposed leaf biomass, infected leaf biomass, and both asexual and sexual spore biomass are zero (E=I=A=S=0), while the susceptible and resistant leaf biomasses remain positive. Setting the time derivatives of the host compartments to zero in the model equations (4) yields the steady-state values for susceptible and resistant leaf biomass:

$$\begin{aligned} H^0 = \frac{\kappa \Lambda }{\mu }, \quad R^0 = \frac{(1 - \kappa ) \Lambda }{\mu }. \end{aligned}$$

Thus, the disease-free equilibrium is

6 $$\begin{aligned} \mathcal {E}^0 = \left( H^0, R^0, E^0, I^0, A^0, S^0 \right) = \left( \frac{\kappa \Lambda }{\mu }, \frac{(1 - \kappa ) \Lambda }{\mu }, 0, 0, 0, 0 \right) . \end{aligned}$$

The total leaf biomass density at the disease-free state is therefore

$$\begin{aligned} N^0 = H^0 + R^0 = \frac{\Lambda }{\mu }. \end{aligned}$$

The disease-persistent (endemic) equilibrium, denoted $\mathcal {E}^* = (H^*, R^*, E^*, I^*, A^*, S^*)$, represents a long-term steady state in which the infection remains persistent within the population and infected and susceptible leaf biomass coexist indefinitely. Identifying the conditions under which this equilibrium exists and is stable enables the prediction of outbreak potential and the evaluation of control interventions in banana plantations.

The BSD-persistent equilibrium is determined by setting the time derivatives in the BSD model (4) and (5) equal to zero. This balances the rate of new infections against the removal of infected biomass and the decay of pathogens. Solving the resulting algebraic system gives

7 $$\begin{aligned}&\mathcal {E}^*=\left\{ \frac{\kappa \Lambda }{\beta (h,T)\Theta _1^*+\mu },\frac{(1-\kappa )\Lambda }{(1-\delta )\beta (h,T)\Theta _1^*+\mu },\frac{\beta (h,T)\Theta _1^*\Theta _2^*}{\Delta _1},\frac{\gamma E^*}{\Delta _2},\frac{\eta \gamma E^*}{\Delta _2\Delta _3}\right. \nonumber \\ ,&\qquad \left. \frac{\alpha \gamma ^2\lambda (E^*)^2}{\Delta _2\Delta _3(\Delta _2+\gamma \lambda E^*)}\right\} , \end{aligned}$$

where

$$\begin{aligned} \Theta _1^*=\theta S^*+\psi \frac{A^*}{N^*}, \,\Theta _2^*=H^*+(1-\delta )R^*,\, \Delta _1=\gamma +\mu ,\,\Delta _2=\mu +\rho , \, \Delta _3=\mu _P+\rho . \end{aligned}$$

Here, $\Delta _2$ denotes the total rate at which infected leaf biomass leaves the infected compartment through spore production, natural senescence, and cultural management, while $\Delta _3$ represents the total loss rate of pathogen spores due to natural mortality and cultural practices.

### $\mathcal {R}_0$ for BSD

The basic reproduction number, denoted $\mathcal {R}_0$, is a threshold parameter in epidemiology that quantifies the expected number of secondary infections produced by a single infected individual introduced into a fully susceptible population. It determines whether an infection can invade and establish itself ($\mathcal {R}_0 > 1$) or is expected to die out ($\mathcal {R}_0 < 1$). Computing $\mathcal {R}_0$ is essential for assessing invasion risk, designing control strategies, and identifying interventions for reducing disease transmission.

A standard and rigorous approach for deriving $\mathcal {R}_0$ is the next-generation matrix method (Diekmann et al. 1990; Driessche and Watmough 2002). This technique constructs two matrices: the transmission Jacobian matrix $\mathcal {F}$, which captures the rate of new infections in each infected compartment, and the transition Jacobian matrix $\mathcal {V}$, which describes the rates of progression or removal among infected compartments. The basic reproduction number is then the spectral radius (dominant eigenvalue) of the next-generation matrix $\mathcal{F}\mathcal{V}^{-1}$.

In our model, the infected compartments are the exposed *E*, the infected *I*, and the asexual spores (conidia), *A*. Sexual spores (ascospores, *S*) are excluded from the computation of $\mathcal {R}_0$. Near the disease-free equilibrium, the infected leaf biomass I≈0, so the mating function satisfies $g(I) I \sim I^2$. Consequently, sexual spore production is a higher-order term that vanishes quadratically and does not contribute to the leading-order invasion dynamics in the linearized system. We therefore consider the vector of infected states $\textbf{X} = (E, I, A)^\top$ and express the dynamics of these compartments as

$$\begin{aligned} \frac{d\textbf{X}}{dt} = F(\textbf{X}) - V(\textbf{X}), \end{aligned}$$

where the new infection matrix is

$$F(\textbf{X}) = \begin{bmatrix} \beta (h,T) \psi \frac{A}{N} \left[ H + (1 - \delta ) R \right] \\ 0 \\ 0 \end{bmatrix},$$

and the transition matrix is

$$V(\textbf{X}) = \begin{bmatrix} (\gamma + \mu ) E \\ - \gamma E + (\mu + \rho ) I \\ - \eta I + (\mu _P + \rho ) A \end{bmatrix}.$$

To compute $\mathcal {R}_0$, evaluate the Jacobians of *F* and *V*, $\mathcal {F}$ and $\mathcal {V}$ respectively, at the disease-free equilibrium $\mathcal {E}^0 = (H^0, R^0, 0, 0, 0, 0)$, where $H^0 = \kappa \Lambda / \mu$ and $R^0 = (1 - \kappa ) \Lambda / \mu$,

$$\begin{aligned} \mathcal {F}(\mathcal {E}^0)&= \begin{bmatrix} 0& 0 & \beta \psi \left[ \kappa \delta +(1-\delta )\right] \\ 0 & 0 & 0\\ 0 & 0 & 0 \end{bmatrix}, \qquad \mathcal {V}(\mathcal {E}^0) = \begin{bmatrix} \gamma +\mu & 0 & 0 \\ -\gamma & \mu +\rho & 0 \\ 0 & -\eta & \mu _P+\rho \end{bmatrix}. \end{aligned}$$

The basic reproduction number is then

$$\begin{aligned} \mathcal {R}_0 = \sigma \bigl (\mathcal {F}\mathcal {V}^{-1} \bigr ), \end{aligned}$$

where $\sigma (\cdot )$ denotes the spectral radius. In our case,

$$\mathcal {V}^{-1}(\mathcal {E}^0) = \begin{bmatrix} \dfrac{1}{\gamma +\mu } & 0 & 0 \\ \dfrac{\gamma }{(\gamma +\mu )(\mu +\rho )}& \dfrac{1}{\mu +\rho }& 0 \\ \dfrac{\eta \gamma }{(\gamma +\mu )(\mu +\rho )(\mu _P+\rho )} & \dfrac{\eta }{(\mu +\rho )(\mu _P+\rho )} & \dfrac{1}{(\mu _P+\rho )} \end{bmatrix}.$$

It is straightforward to see that the matrix product $\mathcal {F V}^{-1}$ has a single nonzero eigenvalue,

8 $$\begin{aligned} \mathcal {R}_0=\frac{\beta (h,T)\psi \eta \gamma [\delta \kappa +(1-\delta )]}{(\gamma +\mu )(\mu _P+\rho )(\mu +\rho )}. \end{aligned}$$

This expression for $\mathcal {R}_0$ reflects the expected number of new infections (primarily via conidia transmission in the early invasion phase) produced by one initially infected unit in a fully susceptible leaf population, accounting for latency, asexual spore production, and decay processes. In regimes where mate limitation is negligible at invasion, i.e., sexual reproduction contributes negligibly near I=0, $\mathcal {R}_0$ is dominated by the asexual transmission pathway. However, the full nonlinear model, including sexual spore production and the associated Allee effect, gives rise to a backward bifurcation, as demonstrated subsequently.

### Disease Prevalence for $\mathcal {R}_0<1$

As mentioned in the previous subsection, the infections produced by ascospores are not captured in the computation of $\mathcal {R}_0$ because, for small infected leaf biomass, the corresponding term of the force of infection is nonlinear, $\alpha g(I)I\sim \alpha \lambda I^2$. Here, we show that the disease can successfully invade a healthy population despite having $\mathcal {R}_0<1$.

Let us denote by Φ the effective host biomass available for infection at the BSD-free equilibrium,

9 $$\begin{aligned} \Phi =\frac{H^0+(1-\delta )R^0}{N^0}=\kappa +(1-\delta )(1-\kappa )=\delta \kappa +(1-\delta ). \end{aligned}$$

During an early invasion phase, disease progression is slow, allowing us to assume that the exposed leaves, conidia, and ascospores are at a quasi-steady state, that is, dE/dt=dA/dt=dS/dt=0. Therefore,

10 $$\begin{aligned} E=\frac{\Gamma \times \left( H^0+(1-\delta )R^0\right) }{\gamma +\mu },\quad A=\frac{\eta I}{\mu _P+\rho },\quad S=\frac{\alpha g(I)I}{\mu _P+\rho }. \end{aligned}$$

Substituting the last two approximations into the force of infection (2) yields

11 $$\begin{aligned} \Gamma =\beta (h,T)\left( \theta \alpha g(I)+\frac{\psi \eta }{N^0}\right) \frac{I}{\mu _P+\rho }. \end{aligned}$$

Substituting the approximation to *E* in the equation for *dI*/*dt* and recalling that $N^0=\Lambda /\mu$ gives

$$\begin{aligned} \frac{1}{I}\frac{dI}{dt}&= \gamma \frac{E}{I} -(\mu +\rho )\\&= \gamma \beta (h,T)\left( \theta \alpha g(I)+\frac{\psi \eta }{N^0}\right) \frac{1}{\mu _P+\rho }\frac{\left( H^0+(1-\delta )R^0\right) }{\gamma +\mu }-(\mu +\rho )\\&=(\gamma \beta (h,T) \theta \alpha g(I) N^0+\gamma \beta (h,T)\psi \eta )\frac{\Phi }{(\mu _P+\rho )(\gamma +\mu )}-(\mu +\rho ) \\&=\underbrace{\frac{\Delta \theta \alpha g(I)}{\mu _P+\rho }}_{NL}+\underbrace{(\mathcal {R}_0-1)(\mu +\rho )}_{L}, \end{aligned}$$

where

$$\begin{aligned} \Delta =\frac{\beta (h,T)\Lambda \gamma \Phi }{\mu (\gamma +\mu )} \end{aligned}$$

and the nonlinear and linear terms of the equation are denoted by *NL* and *L*, respectively. If the nonlinear term contribution is neglected, because *I* is very small, then all the growth of infected biomass comes from the linear contribution when L>0, or equivalently, when $\mathcal {R}_0>1$, as expected.

Let us now consider the effects of the nonlinear term *NL* and ask whether it is possible to have a disease invasion even if $\mathcal {R}_0<1$. That would be the case if the inequality

12 $$\begin{aligned} g(I)>\underbrace{\frac{(1-\mathcal {R}_0)(\mu +\rho )(\mu _P+\rho )}{\Delta \theta \alpha }}_{D} \end{aligned}$$

is satisfied. By recalling the definition of *g*(*I*) given in (3), we obtain the following equivalent condition for a successful disease invasion

13 $$\begin{aligned} I \,\lambda \left( \frac{1}{D}-1\right) >1, \end{aligned}$$

from which it follows that *D* must be less than one. Let $I_c$ be the invasion threshold, i.e., the value of *I* at which equality is held in the previous relationship. Here, *D* denotes the threshold function governing the existence of biologically feasible endemic equilibria. The sign of *D* determines whether one, two, or no positive endemic equilibria exist, thereby identifying the parameter region in which backward bifurcation may occur. Then

14 $$\begin{aligned} I_c=\frac{D}{\lambda (1-D)}. \end{aligned}$$

In conclusion, when $\mathcal {R}_0<1$ but $I>I_c$ and D<1, there will be disease invasion. During the invasion phase, the pathogen is introduced into a largely disease-free plantation, and infected biomass remains very small. Under these conditions, encounters between compatible mating types are rare, so sexual reproduction is strongly suppressed by mate limitation. Consequently, pathogen establishment is governed primarily by the linear (*L*) component of the model, corresponding to asexual spore production and transmission. As the infected biomass increases, mating opportunities become more frequent, activating the nonlinear (*NL*) component associated with density-dependent sexual reproduction. The transition between these two regimes explains why the dynamics differ between early invasion and established epidemics.

The critical infected biomass $I_c$ represents the threshold above which nonlinear sexual reproduction becomes sufficiently strong to contribute substantially to pathogen persistence. Therefore, while $\mathcal {R}_0$ characterizes the ability of the pathogen to invade a disease-free plantation through the dominant linear transmission pathway, $I_c$ quantifies the biomass level required for nonlinear reproductive mechanisms to sustain endemic transmission. This distinction underlies the backward bifurcation observed in the model, where disease persistence may occur even when $\mathcal {R}_0<1$.

The biological interpretation of these conditions is that the nonlinear effects of sexual production of ascospores may trigger an outbreak to advance when the threshold of infected biomass $I_c$ is crossed, and ascospore production is sufficiently rapid. This suggests bistability in the model via a backward bifurcation, which is a subcritical transcritical bifurcation.

### The Backward Bifurcation

This resilience of the BSD can be better understood by considering a backward bifurcation, in which the disease can maintain stable endemic states even when the basic reproduction number $\mathcal {R}_0$ falls below 1, defying conventional thresholds for eradication. In our model, this arises from dual spore transmission pathways and mate limitation in ascospore production, leading to bistability and hysteresis that complicate control strategies. The rigorous analysis draws on Theorem 4.1 from Castillo-Chavez and Song (2004), originally applied to tuberculosis dynamics, to confirm subcritical behavior and underscore the need for **conventional and effective disease management strategies**, such as resistant varieties.

#### Numerical Results

**Fig. 5:**
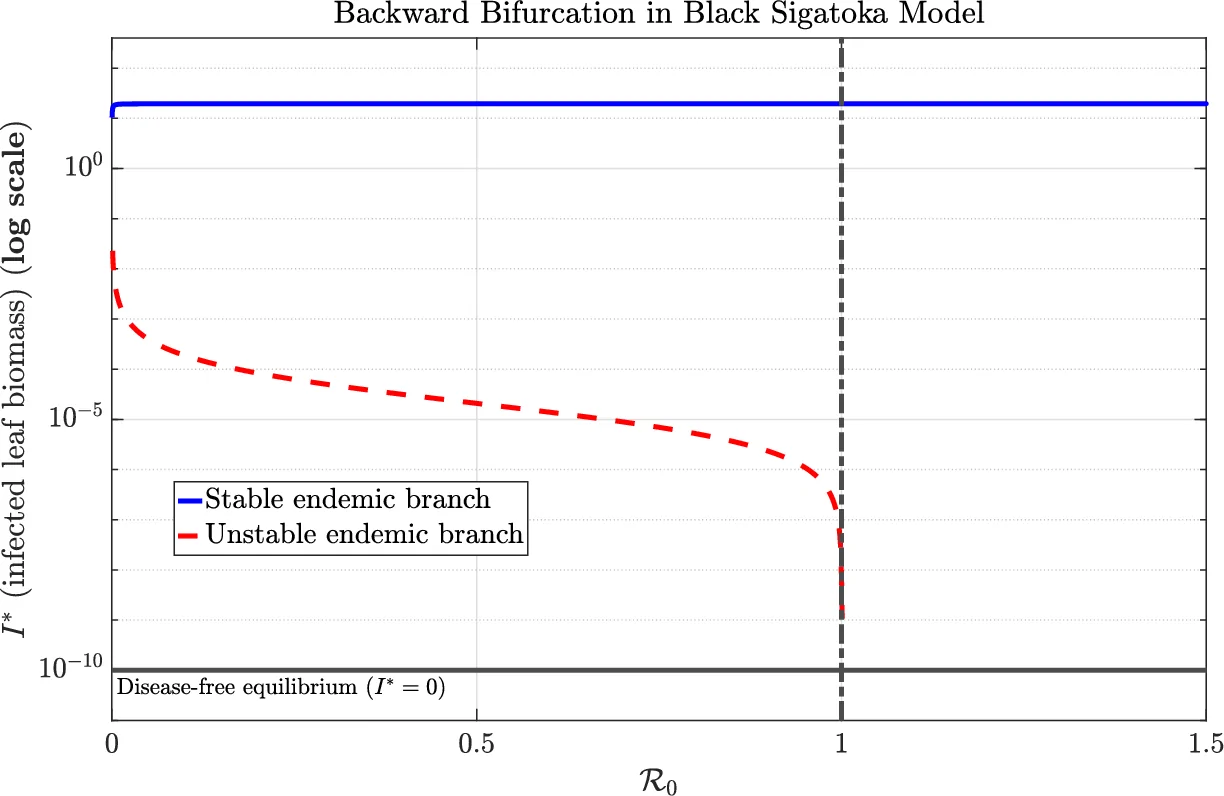
Backward bifurcation in the model (4). The stable endemic branch (blue) extends beyond $\mathcal {R}_0=1$. The unstable branch (red dashes) emerges at $\mathcal {R}_0=1$ and bends “backward". The coexistence of the positive and disease-free equilibria for $\mathcal {R}_0<1$ persists for extremely small values of $\mathcal {R}_0$. The fold on the bifurcation curve for small values of $\mathcal {R}_0$ is not displayed due to computational limitations (color figure online)

Here, we present numerical computations of the equilibria for the model given in Eq. (4), using the parameter values in Table 2. The results, illustrated in Fig. 5, clearly show the occurrence of a backward bifurcation.

In the context of our BSD model, the basic reproductive number, $\mathcal {R}_0$, is an inadequate predictor of disease eradication. While the classical threshold theory suggests that reducing $\mathcal {R}_0$ below one is sufficient for elimination, the biology of *Mycosphaerella fijiensis* supports a more complex dynamic. The pathogen spreads through two complementary transmission pathways: asexual conidia, which drive rapid local reinfection within plantations, and sexually produced ascospores, which facilitate long-distance dispersal and the establishment of new infection foci. Coupled with density-dependent sexual reproduction, these dual spore pathways promote pathogen persistence and may allow the pathogen to persist on a stable branch even when $\mathcal {R}_0<1$ due to the bistability associated with the backward bifurcation. The bistable behavior arises predominantly from the nonlinear dynamics in the sexual reproduction cycle, which creates an unstable equilibrium branch in the bifurcation diagram (red dashes).

The bifurcation diagram in Fig. 5 shows two remarkable characteristics. First, the critical value of $\mathcal {R}_0$, for which the fold in the bifurcation curve occurs, is exceptionally low. This indicates that outbreaks of Black Sigatoka disease can emerge under a wide range of epidemiological conditions, even when the basic reproduction number is only slightly above zero. Second, the unstable equilibrium branch remains extremely close to zero for very small positive values of $\mathcal {R}_0$. Consequently, the introduction of even a tiny initial density of infected leaf biomass may be sufficient to trigger a full epidemic outbreak, as the system would lie within the basin of attraction of the stable endemic equilibrium. The importance of this result lies in its implication for disease management: reducing $\mathcal {R}_0$ below unity alone may not eliminate BSD. Instead, control measures must also reduce infected biomass below the unstable threshold to shift the system into the basin of attraction of the disease-free equilibrium.

These observations are consistent with the fact that BSD has not been successfully eradicated in practice, but, on a single occasion in Australia between 2001 and 2005, following an outbreak in the Tully region of Queensland (Peterson et al. 2005; Cook et al. 2013). The program required an aggressive approach that included the complete destruction of infected plantations, the removal and burial of plant materials, the burning of infected leaves, strict quarantine zones, surveillance, and movement controls coordinated by Biosecurity Queensland (Henderson et al. 2006; Sosnowski et al. 2009). The Tully Banana Black Sigatoka Eradication program involved the destruction of approximately 95% of banana plantations in the infected zone and a six-month ban on replanting to ensure the pathogen had no living host to maintain its population (Henderson et al. 2006). This is consistent with our model results, which suggest that successful management depends on intensive biomass reduction to shift the system into the narrow basin of the stable state, representing a BSD-free equilibrium.

#### Analytical Results

Here, we analytically investigate the presence of a backward bifurcation in the BSD model (4). We follow Castillo-Chavez and Song (2004), Theorem 4.1, which provides conditions, derived from eigenvectors and second-order partial derivatives, under which a dynamical system undergoes a backward bifurcation at the disease-free equilibrium, when the bifurcation parameter $\beta _0$ passes through $\beta _0^*$, where $\beta _0^*$ is the value that corresponds to $\mathcal {R}_0=1$.

##### Theorem 3.1

Let us denote $\textbf{x}=(H,R,E,I,A,S)$ and $\textbf{f}=d\textbf{x}/dt$. The bifurcation coefficients $\textbf{a}$ and $\textbf{b}$ are defined as

$$\begin{aligned} \textbf{a}=\sum _{k,i,j=1}^{6}v_kw_iw_j\frac{\partial ^2f_k(\mathcal {E}^0,\beta _0^*)}{\partial x_i\partial x_j},\qquad \textbf{b}=\sum _{k,i=1}^{6}v_kw_i\frac{\partial ^2f_k(\mathcal {E}^0,\beta _0^*)}{\partial x_i\partial \beta _0^*}. \end{aligned}$$

Then, the BSD model (4) undergoes a backward bifurcation at $\beta _0=\beta _0^*$, i.e. $\mathcal {R}_0=1$, when $\textbf{a}>0$, $\textbf{b}>0$ and $\lambda >\lambda ^{\text {crit}}$, where

15 $$\begin{aligned} \lambda ^{\text {crit}}=\frac{\beta _0^*\Pi \mu \psi ^2\eta ^2[(1-\delta )+\delta \kappa (2+\delta )]}{\alpha \theta \Lambda ^2(\mu _P+\rho )[\delta \kappa +(1-\delta )]}. \end{aligned}$$

##### Proof

Setting $\mathcal {R}_0=1$ and solving for $\beta _0^*$ in (8) gives;

16 $$\begin{aligned} \beta _0^*=\frac{(\gamma +\mu )(\mu _P+\rho )(\mu +\rho )}{\Pi \psi \eta \gamma [\delta \kappa +(1-\delta )]}, \end{aligned}$$

where $\Pi =\frac{h}{h+K_h}\exp {\left( -\frac{(T-\hat{T})^2}{2\sigma _T^2}\right) }$. The Jacobian matrix of the BSD model (4) evaluated at the BSD-free equilibrium, $\mathcal {E}^0$ with $\beta _0=\beta _0^*$ is

17 $$\begin{aligned} \mathcal {J}(\mathcal {E}^0,\beta _0^*)= \begin{bmatrix} -\mu & 0& 0& 0& -\beta _0^*\Pi \psi \kappa & -\frac{\beta _0^*\Pi \theta \kappa \Lambda }{\mu }\\ 0& -\mu & 0& 0& -\beta _0^*\Pi \psi (1-\delta )(1-\kappa )& -\frac{\beta _0^*\Pi \theta \Lambda (1-\delta )(1-\kappa )}{\mu }\\ 0& 0& -(\gamma +\mu )& 0& \beta _0^*\Pi \psi [\delta \kappa +(1-\delta )]& \frac{\beta _0^*\Pi \theta \Lambda [\delta \kappa +(1-\delta )]}{\mu }\\ 0& 0& \gamma & -(\mu +\rho )& 0& 0\\ 0& 0& 0& \eta & -(\mu _P+\rho )& 0\\ 0& 0& 0& 0& 0& -(\mu _P+\rho ) \end{bmatrix}. \end{aligned}$$

This matrix has $\lambda _1=\lambda _2=-\mu$, and $\lambda _6=-(\mu _P+\rho )$ as eigenvalues. The remaining eigenvalues are obtained from the sub-matrix

18 $$\begin{aligned} \hat{\mathcal {J}}(\mathcal {E}^0,\beta _0^*)= \begin{bmatrix} -(\gamma +\mu )& 0& \beta _0^*\Pi \psi [\delta \kappa +(1-\delta )]\\ \gamma & -(\mu +\rho )& 0\\ 0& \eta & -(\mu _P+\rho ) \end{bmatrix}, \end{aligned}$$

for which the associated characteristic polynomial at χ is

19 $$\begin{aligned} |\chi \textbf{I}-\hat{\mathcal {J}}(\mathcal {E}^0,\beta _0^*)|=(\chi +\gamma +\mu )(\chi +\mu +\rho )(\chi +\mu _P+\rho )-\beta _0^*\Pi \psi \gamma \eta [\delta \kappa +(1-\delta )]. \end{aligned}$$

At χ=0, there is an eigenvalue when

$$\begin{aligned} (\gamma +\mu )(\mu +\rho )(\mu _P+\rho )-\beta _0^*\Pi \psi \gamma \eta [\delta \kappa +(1-\delta )]&=0,\\ \frac{\beta _0^*\Pi \psi \gamma \eta [\delta \kappa +(1-\delta )]}{(\gamma +\mu )(\mu +\rho )(\mu _P+\rho )}&=1, \mathcal {R}_0=1. \end{aligned}$$

Therefore, at $\beta _0=\beta _0^*$ that is $\mathcal {R}_0=1$, the Jacobian matrix associated with the BSD model (4) evaluated at $\mathcal {E}^0$ has a simple zero eigenvalue (with all other eigenvalues having negative real part). Thus, the Jacobian matrix has a right eigenvector given by $\textbf{w}=(w_1,w_2,w_3,w_4,w_5,w_6)$ and a left eigenvector given by $\textbf{v}=(v_1,v_2,v_3,v_4,v_5,v_6)$ corresponding to the zero eigenvalue. A right eigenvector satisfies

20 $$\begin{aligned} \begin{bmatrix} -\mu & 0& 0& 0& -\beta _0^*\Pi \psi \kappa & -\frac{\beta _0^*\Pi \theta \kappa \Lambda }{\mu }\\ 0& -\mu & 0& 0& -\beta _0^*\Pi \psi (1-\delta )(1-\kappa )& -\frac{\beta _0^*\Pi \theta \Lambda (1-\delta )(1-\kappa )}{\mu }\\ 0& 0& -(\gamma +\mu )& 0& \beta _0^*\Pi \psi [\delta \kappa +(1-\delta )]& \frac{\beta _0^*\Pi \theta \Lambda [\delta \kappa +(1-\delta )]}{\mu }\\ 0& 0& \gamma & -(\mu +\rho )& 0& 0\\ 0& 0& 0& \eta & -(\mu _P+\rho )& 0\\ 0& 0& 0& 0& 0& -(\mu _P+\rho ) \end{bmatrix} \begin{bmatrix} w_1\\ w_2\\ w_3\\ w_4\\ w_5\\ w_6 \end{bmatrix} = \begin{bmatrix} 0\\ 0\\ 0\\ 0\\ 0\\ 0 \end{bmatrix} \end{aligned}$$

Solving for $w_1$, $w_2$, $w_3$, and $w_5$ in terms of $w_4$ yields

$$\begin{aligned} w_1&=-\frac{\beta _0^*\Pi \psi \kappa \eta }{\mu (\mu _P+\rho )}w_4,\;\; w_2=-\frac{\beta _0^*\Pi \psi \eta (1-\delta )(1-\kappa )}{\mu (\mu _P+\rho )}w_4,\;\; w_3=\frac{\mu +\rho }{\gamma }w_4,\\ w_4&>0,\;\; w_5=\frac{\eta }{\mu _P+\rho }w_4,\;\; w_6=0. \end{aligned}$$

A left eigenvector satisfies

21 $$\begin{aligned} &  \begin{bmatrix} -\mu & 0& 0& 0& 0& 0\\ 0& -\mu & 0& 0& 0& 0\\ 0& 0& -(\gamma +\mu )& \gamma & 0& 0\\ 0& 0& 0& -(\mu +\rho )& \eta & 0\\ -\beta _0^*\Pi \psi \kappa & -\beta _0^*\Pi \psi (1-\delta )(1-\kappa )& \beta _0^*\Pi \psi [\delta \kappa +(1-\delta )]& 0& -(\mu _P+\rho )& 0\\ -\frac{\beta _0^*\Pi \theta \kappa \Lambda }{\mu }& -\frac{\beta _0^*\Pi \theta \Lambda (1-\delta )(1-\kappa )}{\mu }& \frac{\beta _0^*\Pi \theta \Lambda [\delta \kappa +(1-\delta )]}{\mu }& 0& 0& -(\mu _P+\rho ) \end{bmatrix} \nonumber \\ &  \quad \begin{bmatrix} v_1\\ v_2\\ v_3\\ v_4\\ v_5\\ v_6 \end{bmatrix} = \begin{bmatrix} 0\\ 0\\ 0\\ 0\\ 0\\ 0 \end{bmatrix} \end{aligned}$$

Solving for $v_4$, $v_5$, and $v_6$ in terms of $v_3$ gives

$$\begin{aligned} v_1&=0,\; v_2=0,\; v_3>0,\;\; v_4=\frac{\gamma +\mu }{\gamma }v_3,\;\; v_5=\frac{(\mu +\rho )(\gamma +\mu )}{\eta \gamma }v_3, \\&\quad v_6=\frac{\beta _0^*\Pi \theta \Lambda [\delta \kappa +(1-\delta )]}{\mu (\mu _P+\rho )}v_3. \end{aligned}$$

Since $v_1$ and $v_2$ are zero, we only compute the second-order partial derivatives of $f_3$, $f_4$, $f_5$, and $f_6$,

$$\begin{aligned} \frac{\partial ^2f_3(\mathcal {E}^0,\beta _0^*)}{\partial H\partial S}&=\frac{\partial ^2f_3(\mathcal {E}^0,\beta _0^*)}{\partial S\partial H}=\beta _0^*\Pi \theta ,\\ \frac{\partial ^2f_3(\mathcal {E}^0,\beta _0^*)}{\partial H\partial A}&=\frac{\partial ^2f_3(\mathcal {E}^0,\beta _0^*)}{\partial A\partial H}=\frac{\beta _0^*\Pi \psi \mu }{\Lambda },\\ \frac{\partial ^2f_3(\mathcal {E}^0,\beta _0^*)}{\partial R\partial S}&=\frac{\partial ^2f_3(\mathcal {E}^0,\beta _0^*)}{\partial S\partial R}=\beta _0^*\Pi \theta (1-\delta ),\\ \frac{\partial ^2f_3(\mathcal {E}^0,\beta _0^*)}{\partial R\partial A}&=\frac{\partial ^2f_3(\mathcal {E}^0,\beta _0^*)}{\partial A\partial R}=\frac{\beta _0^*\Pi \psi \mu (1-\delta )}{\Lambda },\\ \frac{\partial ^2f_6(\mathcal {E}^0,\beta _0^*)}{\partial I^2}&=2\alpha \lambda ,\\ \frac{\partial ^2f_3(\mathcal {E}^0,\beta _0^*)}{\partial A\partial \beta _0^*}&=\Pi \psi \mu [\delta \kappa +(1-\delta )],\\ \frac{\partial ^2f_3(\mathcal {E}^0,\beta _0^*)}{\partial S\partial \beta _0^*}&=\frac{\Pi \theta \Lambda [\delta \kappa +(1-\delta )]}{\mu }. \end{aligned}$$

Using these expressions in the definitions of $\textbf{a}$ gives

22 $$\begin{aligned} \textbf{a}=2v_3w_5\frac{\beta _0^*\Pi \psi \mu }{\Lambda }\left[ w_1+(1-\delta )w_2\right] +2v_6w_4^2\alpha \lambda . \end{aligned}$$

Substituting the values of $w_1$, $w_2$, $w_5$, and setting $w_4=1$ yields

23 $$\begin{aligned} \textbf{a}=\frac{2v_3\alpha \lambda \beta _0^*\Pi \theta \Lambda [\delta \kappa +(1-\delta )]}{\mu (\mu _P+\rho )}-\frac{2v_3(\beta _0^*\Pi \psi \eta )^2[(1-\delta )+\delta \kappa (2+\delta )]}{\Lambda (\mu _P+\rho )}. \end{aligned}$$

Therefore, if

24 $$\begin{aligned} \lambda >\underbrace{\frac{\beta _0^*\Pi \mu \psi ^2\eta ^2[(1-\delta )+\delta \kappa (2+\delta )]}{\alpha \theta \Lambda ^2(\mu _P+\rho )[\delta \kappa +(1-\delta )]}}_{\lambda ^{\text {crit}}} \end{aligned}$$

then $$\textbf{a}>0$$. Moreover,

25 $$\begin{aligned} \textbf{b}=v_3w_5\Pi \psi [\delta \kappa +(1-\delta )]=\frac{\Pi \psi \eta [\delta \kappa +(1-\delta )]}{\mu _P+\rho }v_3>0. \end{aligned}$$

By applying Theorem 4.1 of Castillo-Chavez and Song, we conclude that the BSD model (4) undergoes a backward bifurcation. □

Condition (24) means that the disease can persist at low values of $\mathcal {R}_0$ because the high biomass of infected leaves in an established epidemic overcomes the initial difficulty of mating spores. Using the parameter values from Table 2, we have $\textbf{a}=1.4700\times 10^7$ and $\lambda >\lambda ^{\text {crit}}=0.0011$. This indicates that sexual reproduction of the pathogen occurs well above the threshold required to induce backward bifurcation. Since $\lambda >\lambda ^{\text {crit}}$, the mate limitation in ascospores is a powerful driver of the epidemic’s persistence. Thus, the Black Sigatoka Disease may persist even when $\mathcal {R}_0<1$, and reducing $\mathcal {R}_0$ below unity may not guarantee disease eradication.

## Sensitivity Analysis

Sensitivity analysis is an analytical tool used to quantify the relative impacts of uncertainties in the input parameters for endemic-infected leaf biomass ($I^*$) and their subsequent effects on disease spread. Thus, sensitivity analysis can determine how input variability affects output variability.

### Local Sensitivity Analysis Using the Normalized Forward Sensitivity Index

To quantify the effect of parameter perturbations on $I^*$, we use the normalized forward sensitivity index introduced by Chitnis et al. (2008). For a parameter ξ, the index is defined by:

26 $$\begin{aligned} \Upsilon _{\xi }^{I^*}=\frac{\partial I^*}{\partial \xi }\times \frac{\xi }{I^*}. \end{aligned}$$

This gives the relative change in $I^*$ per unit relative change in the parameter ξ. A positive index indicates that $I^*$ increases as the parameter value increases, whereas a negative index indicates that $I^*$ decreases as the parameter value increases. This indicates that parameters with negative indices may mitigate disease spread in the population as their values increase.

**Fig. 6:**
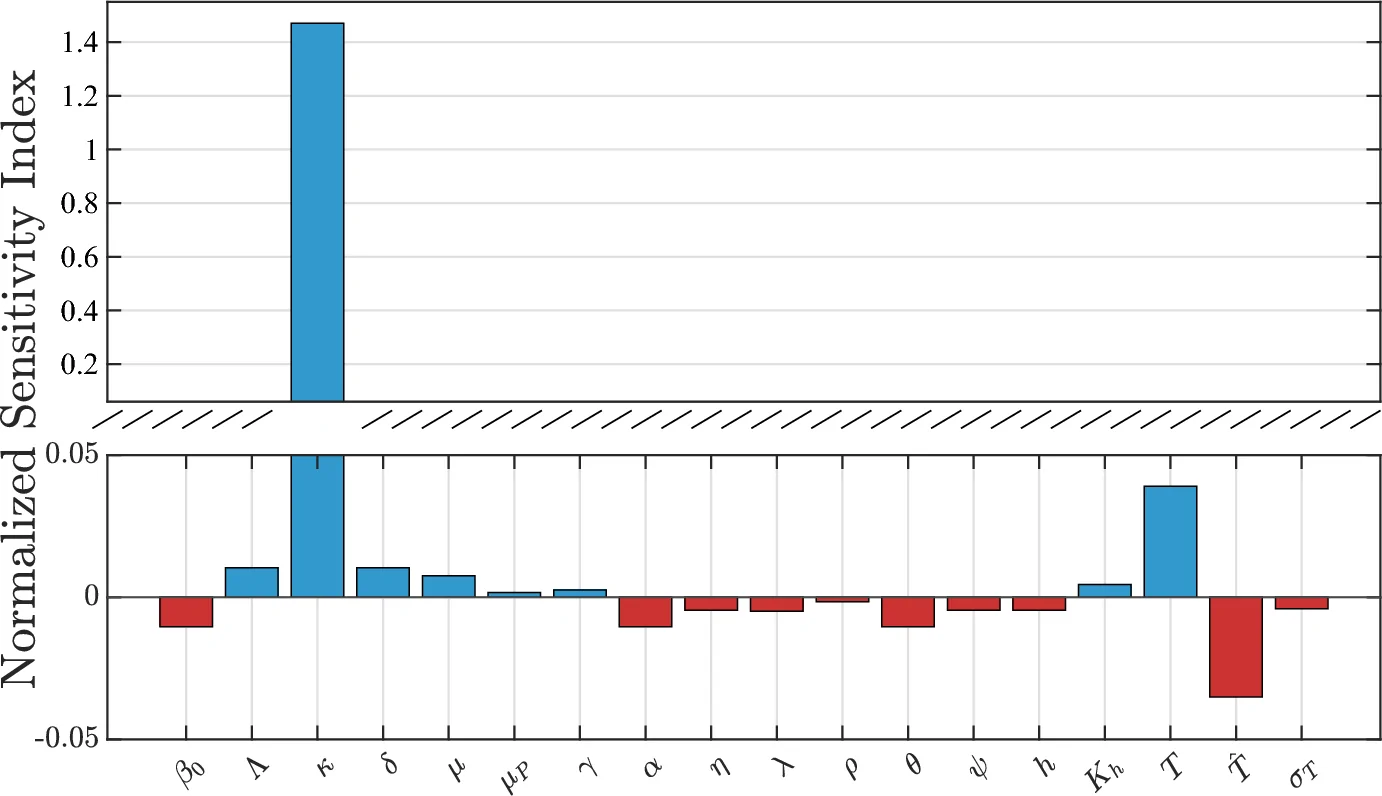
The normalized forward sensitivity indices of the parameters in the endemic-infected leaf biomass ($I^*$) generated using the parameter values stated in Table 2 (color figure online)

Figure 6 shows that the fraction of newly recruited susceptible leaves, κ, is the most influential parameter with an index of 1.47. Thus, a 1% increase in the fraction of newly recruited susceptible leaves leads to a 1.47% increase in $I^*$. In contrast, parameters such as $\beta _0$, Λ, ρ, $\mu _P$, μ, and other parameters in $I^*$ have indices close to zero, indicating weak local influence near the baseline parameter set. Thus, the recruitment structure and host susceptibility drive the $I^*$ far more strongly than most other biological processes do.

### Global Sensitivity Analysis Using the Partial Rank Correlation Coefficient (PRCC)

To assess the global influence of model parameters on the endemic-infected leaf biomass $I^*$, we use Partial Rank Correlation Coefficients (PRCCs) combined with Latin Hypercube Sampling (LHS). This approach evaluates the monotonic relationship between each parameter and $I^*$ while accounting for simultaneous variations in all other parameters. PRCC values close to 1 (-1) indicate a strong positive (negative) global influence on $I^*$, whereas values near zero indicate weak or negligible effects.

**Fig. 7:**
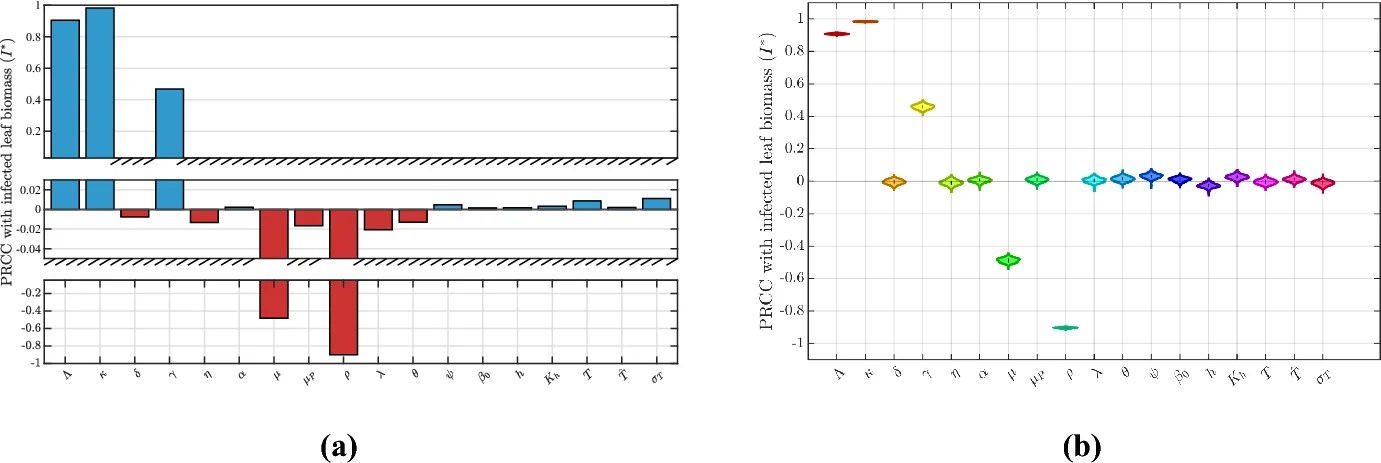
The Partial Rank Correlation Coefficients (PRCCs) results capturing the global sensitivity indices of the parameters in the infected biomass equilibrium ($I^*$). The Violin plot of the Partial Rank Correlation Coefficients (PRCCs) values for the parameters in $I^*$ (Generated distribution around PRCC values.) Generated using the parameter values stated in Table 2 (color figure online)

Figure 7a, the host recruitment parameters Λ and κ have the strongest positive correlations, indicating that $I^*$ is primarily driven by the continuous supply of susceptible leaf biomass. The progression rate γ also has a moderate global positive effect on $I^*$. In contrast, the cultural practices parameter ρ shows a negative correlation, indicating that removing infected biomass is the most effective control mechanism. The natural leaf decay rate μ also contributes negatively by reducing available host biomass.

The violin plots in Fig. 7b illustrate the robustness of these results. Parameters such as Λ, κ, γ, ρ, and μ display narrow distributions centered away from zero, indicating consistent and statistically stable effects across simulations, whereas parameters with distributions concentrated near zero exhibit minimal influence. The results confirm that host recruitment dynamics and cultural practices dominate the global behavior of $I^*$ in the BSD model (4).

## Stochastic Modeling Using the Gillespie Algorithm

To incorporate intrinsic randomness in disease transmission, we formulate a stochastic version of the BSD model using the Gillespie Algorithm, also known as the Stochastic Simulation Algorithm (SSA), Gillespie (1977). In this framework, the biomass variables are treated as discrete “leaf units" or effective leaf units, so that the stochastic process approximates plantation-scale dynamics while retaining the event-by-event structure of the SSA. Although leaf biomass is continuous in reality, this discretization provides a reasonably effective approximation for finite host populations and enables the quantification of demographic stochasticity near eradication thresholds, where deterministic models may fail to capture variability accurately. In this work, one leaf unit represents an effective discrete unit of host biomass rather than an individual physical leaf. This discretization is adopted solely to satisfy the event-based formulation of the Gillespie SSA while preserving the relative biomass dynamics of the deterministic model. The emphasis of the stochastic analysis is on the qualitative effects of demographic stochasticity on disease persistence and extinction rather than on absolute leaf counts.

The stochastic model tracks the state of the vector process $\textbf{Y}=(H, R, E, I, A, S)$, where leaf and spore biomasses are represented as in the previous section. The system’s evolution is governed by a set of discrete events, each associated with a propensity function $a_i(\textbf{Y})$ that defines the probability of occurrence in an infinitesimal time interval, and a corresponding stoichiometric vector $\nu _i$. The full list of events and propensities is provided in Table 3.

At each step, the Gillespie algorithm computes the total propensity, determines the time to the next event, and selects which event occurs based on probabilistic sampling. The system is then updated accordingly, producing stochastic trajectories that reflect variability in disease dynamics. This framework enables comparison with deterministic predictions and provides insight into fluctuations, persistence, and eradication behavior in the presence of stochastic effects.

**Table 3:** List of events, propensity functions, and state changes used in the stochastic simulation of Black Sigatoka Disease dynamics using the Gillespie algorithm

*i*	Event	Propensity function $a_i(\textbf{Y})$	State change $\nu _i$
1	Recruitment of susceptible leaves	$\kappa \Lambda$	H→H+1
2	Recruitment of resistant leaves	$(1 - \kappa )\Lambda$	R→R+1
3	Natural decay of susceptible leaves	μH	H→H-1
4	Natural decay of resistant leaves	μR	R→R-1
5	Infection of susceptible leaves via asexual spores	$\beta (h,T)\psi \frac{A}{N}H$	H→H-1
		E→E+1	
6	Infection of susceptible leaves via sexual spores	$\beta (h,T)\theta SH$	H→H-1
		E→E+1	
7	Infection of resistant leaves via asexual spores	$(1-\delta )\beta (h,T)\psi \frac{A}{N}R$	R→R-1
		E→E+1	
8	Infection of resistant leaves via sexual spores	$(1-\delta )\beta (h,T)\theta SR$	R→R-1
		E→E+1	
9	Progression from exposed to infected	γE	E→E-1
		I→I+1	
10	Natural decay of exposed leaves	μE	E→E-1
11	Natural decay of infected leaves	$(\mu +\rho )I$	I→I-1
12	Asexual spore production	ηI	A→A+1
13	Sexual spore production	$\alpha \frac{\lambda I}{1+\lambda I}I$	S→S+1
14	Loss of asexual spores	$(\mu _P+\rho )A$	A→A-1
15	Loss of sexual spores	$(\mu _P+\rho )S$	S→S-1

**Algorithm 1:**
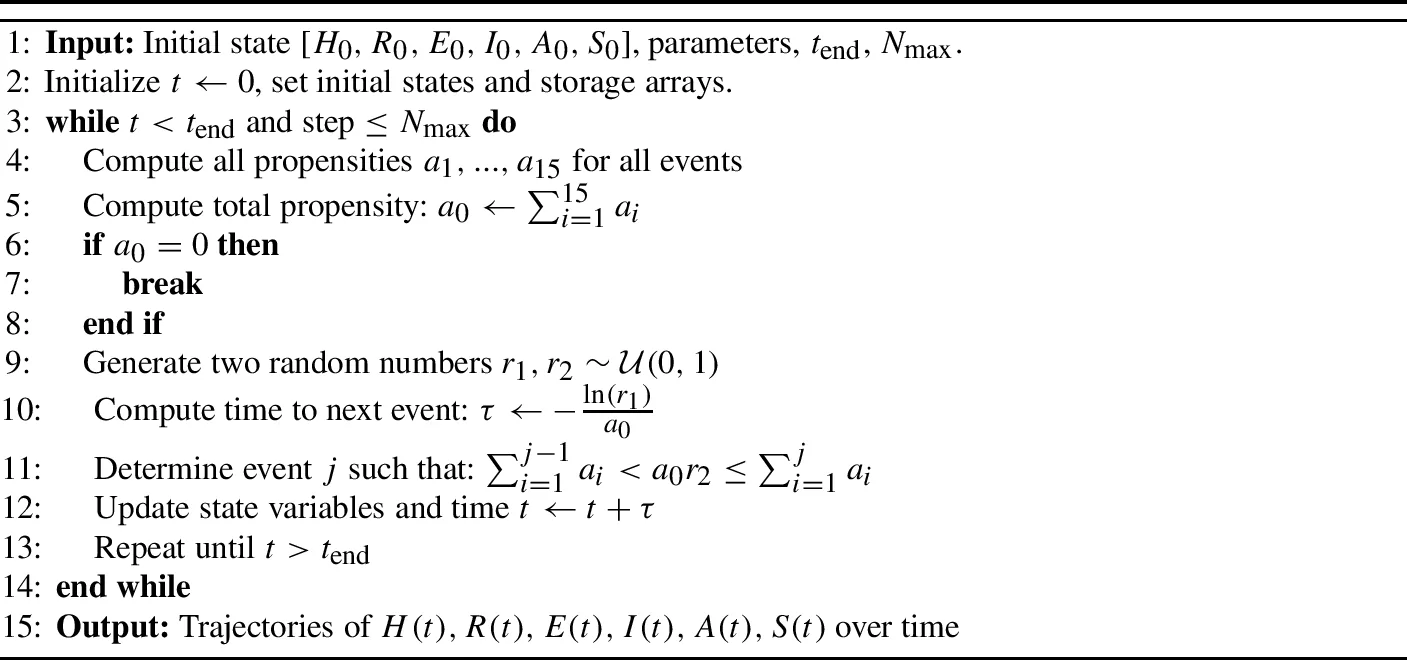
Gillespie algorithm for the BSD model

### Numerical Simulations

We present numerical simulations of deterministic and stochastic BSD models to illustrate disease dynamics, variability, and the probability of disease eradication. Deterministic solutions are obtained from the BSD model (4), while the stochastic realizations are generated using Gillespie SSA.

**Fig. 8:**
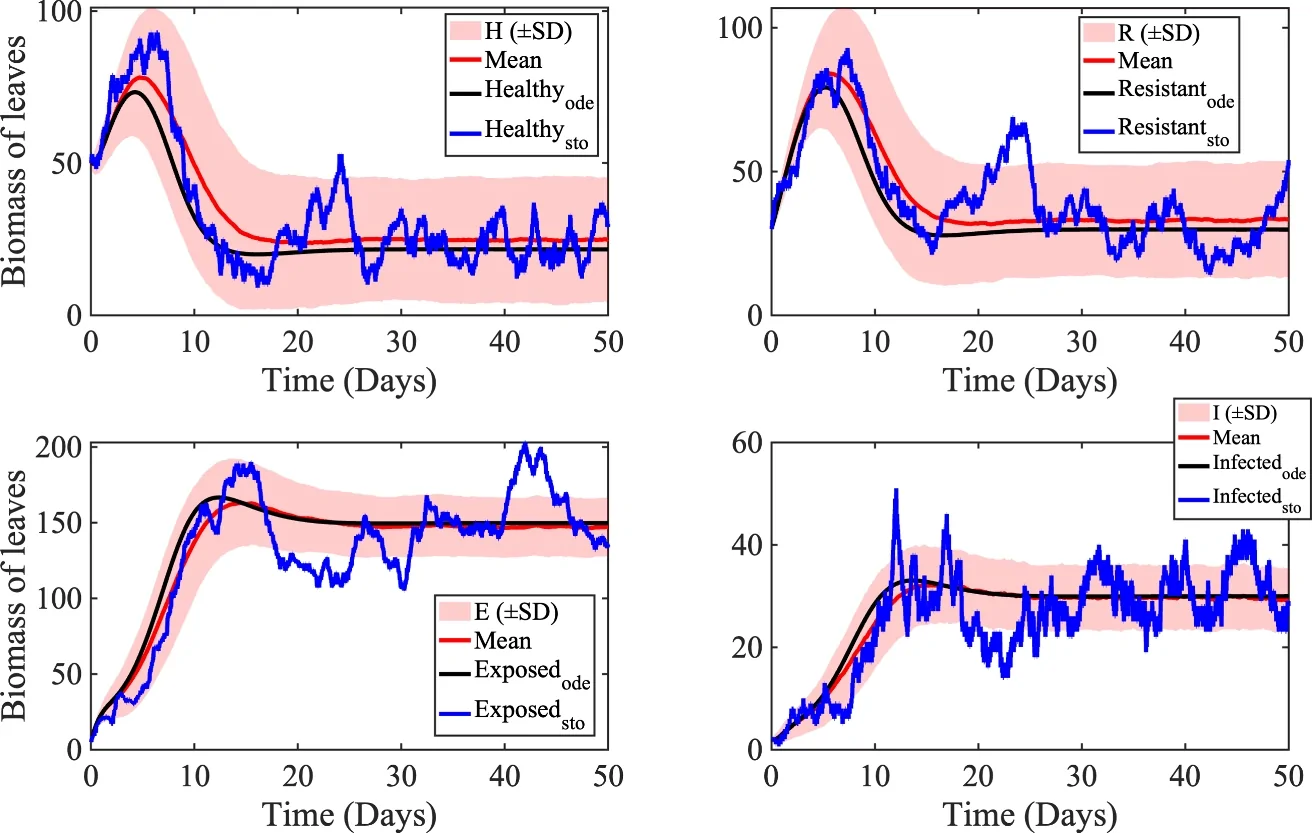
Comparison of deterministic and stochastic simulations of the BSD model over 50 days. Each panel shows the biomass dynamics of a leaf compartment: susceptible leaves *H*(*t*), resistant leaves *R*(*t*), exposed leaves *E*(*t*), and infected leaves *I*(*t*). The black curves represent the deterministic trajectories; the blue curves are a single realization of the stochastic simulation; and the red lines indicate the mean across 400 stochastic realizations. Shaded regions show ±1 standard deviation (SD) from the mean of the stochastic simulations. The stochastic system exhibits larger fluctuations, especially in the early dynamics of *H*(*t*), *R*(*t*), and *I*(*t*), capturing variability not seen in the deterministic counterpart (color figure online)

**Fig. 9:**
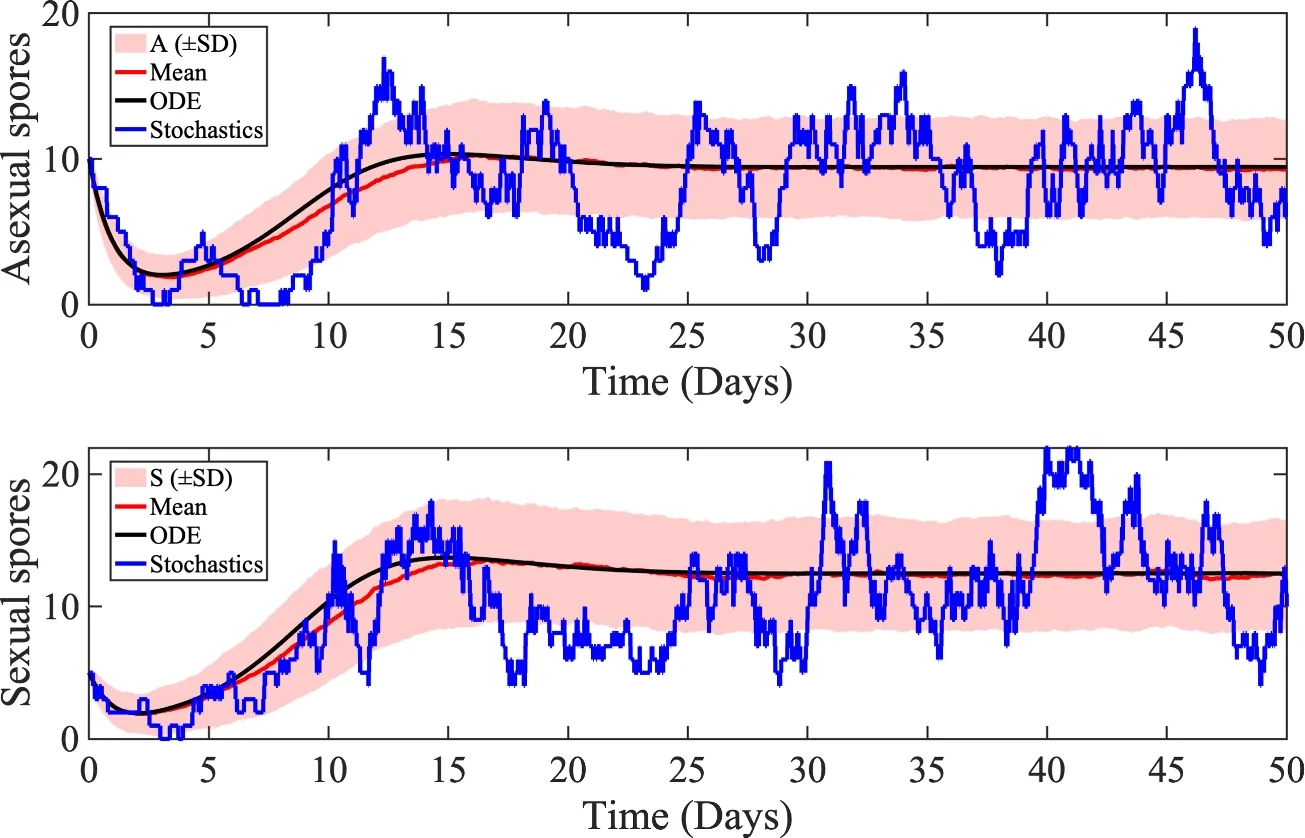
Temporal dynamics of pathogen spores comparing deterministic and stochastic simulations. The top panel shows the biomass of asexual spores *A*(*t*), and the bottom panel displays the biomass of sexual spores *S*(*t*) over 50 days. The asexual spores exhibit moderate stochastic fluctuations, while the sexual spores show highly discrete and burst-like dynamics due to mating constraints at low densities, which are not captured in the smooth ODE solutions (color figure online)

Figures 8 and 9 compare the temporal evolution of leaf and spore biomass under the deterministic and stochastic frameworks, respectively. Stochastic simulations capture variability through individual realizations; the mean across 400 realizations is shown (red curves), together with the corresponding standard deviation. Deterministic trajectories are shown in black. The stochastic paths (blue) exhibit fluctuations that are particularly important during the early stages and at low population levels, reflecting the role of demographic stochasticity. In the leaf compartments (Fig. 8), stochastic effects are most pronounced in the dynamics of susceptible and infected biomass, where variability is highest during transient phases. For the pathogen compartments (Fig. 9), asexual spores show moderate variability, whereas sexual spores display highly intermittent, burst-like behavior due to mate limitation, a feature not captured by the deterministic model. Although the deterministic model predicts persistent disease whenever $\mathcal {R}_0>1$, the stochastic simulations reveal that pathogen extinction remains possible for sufficiently small host populations. This discrepancy arises from demographic stochasticity, which is absent from the deterministic formulation. Consequently, deterministic threshold theory alone may overestimate disease persistence in newly established or heavily managed plantations. The stochastic simulations further demonstrate that the mean trajectory differs systematically from the deterministic solution, particularly for infected biomass and sexual spores. This reflects the nonlinear interaction between mate limitation and demographic stochasticity, illustrating that averaging stochastic trajectories does not necessarily recover deterministic dynamics. A particularly noteworthy observation is the intermittent burst-like production of sexual spores. Unlike the deterministic model, which predicts smooth increases in spore biomass, the stochastic model exhibits episodic reproductive events generated by density-dependent mate limitation. These bursts substantially increase variability during the early stages of invasion and represent a biological phenomenon that cannot be reproduced by deterministic models.

**Fig. 10:**
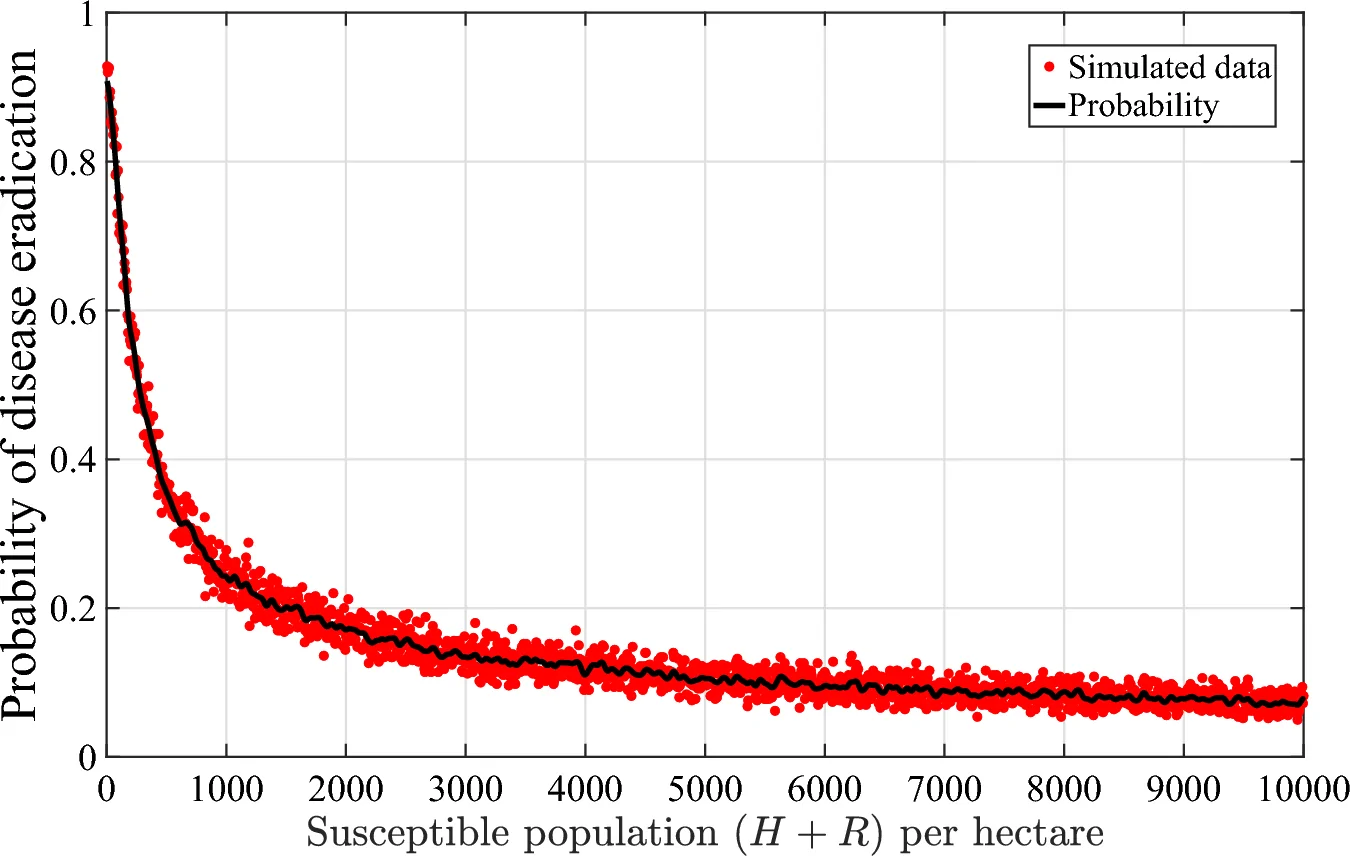
Eradication probability in the model of Black Sigatoka disease as a function of the total initial susceptible and resistant leaf biomass (H(0)+R(0), in leaves per hectare), estimated from 500 stochastic simulations for each initial biomass level. The probability of disease eradication is high when the initial leaf biomass is small (particularly below approximately 1000 leaves per hectare). As the leaf biomass increases, the eradication probability decreases rapidly and stabilizes at a low value, indicating that larger host densities support sustained disease persistence (color figure online)

The probability of disease eradication is approximated in Fig. 10. The plot was obtained by simulating the system across a range of initial host biomass H(0)+R(0)=5,10,⋯,10000. For each population size, 500 independent stochastic realizations were generated over a fixed time horizon of $t_{\max }=100$ days. The initial host composition was chosen such that H(0)=0.6(H(0)+R(0)) and R(0)=0.4(H(0)+R(0)), maintaining a consistent proportion of susceptible and resistant leaves across all simulations, while small initial pathogen populations were fixed at E(0)=1, I(0)=1, A(0)=2, and S(0)=2. A realization is classified as eradicated if the infected class *I*(*t*) reaches zero before the time horizon is reached. The probability of eradication shown in Fig. 10 is computed as the fraction of realizations that lead to eradication across the 500 trials for each initial population size. Each red point represents the estimated probability, while the solid black curve represents a smoothed trend of these values. These results demonstrate that stochastic effects can substantially influence BSD dynamics, particularly in small populations, where they may alter persistence and eradication outcomes relative to deterministic predictions. The stochastic simulations identify a practical host-density threshold below which pathogen eradication becomes increasingly likely. For the parameter values considered here, extinction probability decreases rapidly as total susceptible and resistant biomass increases, suggesting that intensive sanitation capable of reducing infected biomass below this threshold may promote local disease elimination even under conditions where deterministic theory predicts persistence.

**Fig. 11:**
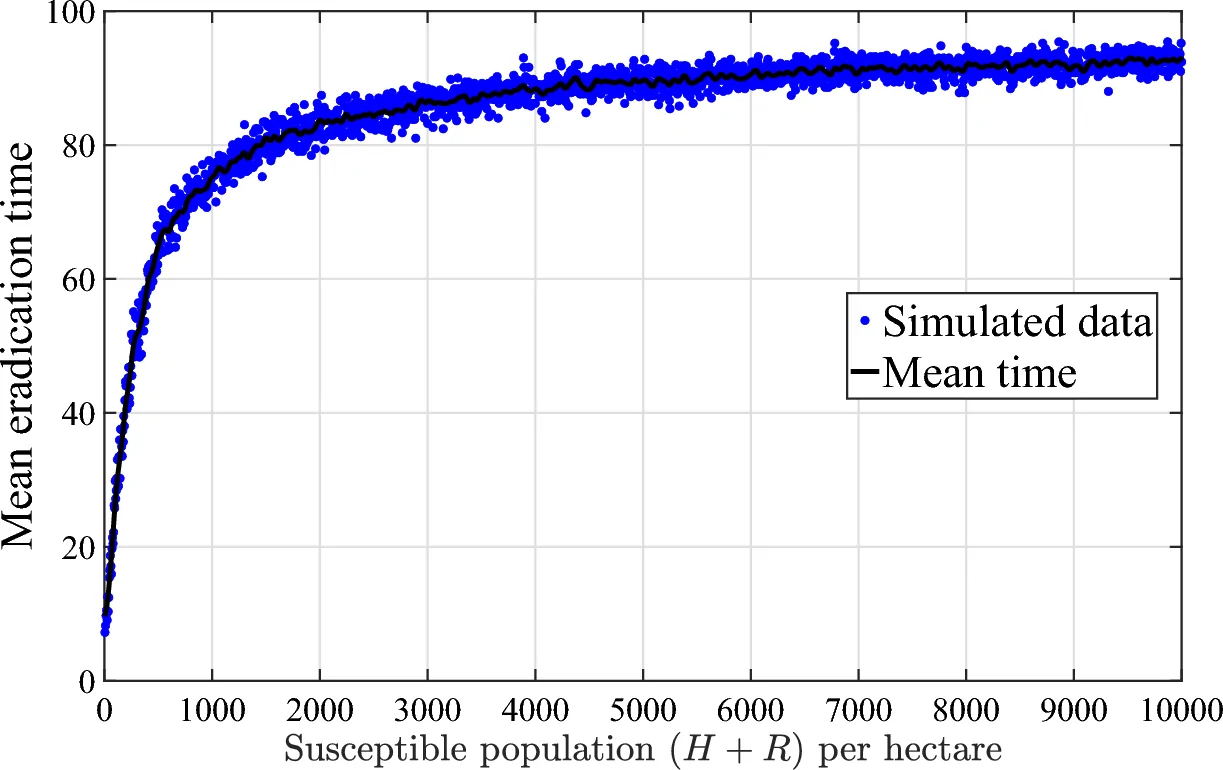
Mean time to disease eradication in the model of Black Sigatoka disease as a function of the total initial susceptible and resistant leaf biomass (H(0)+R(0), in leaves per hectare), estimated from 500 stochastic simulations for each initial biomass level. As the initial leaf biomass increases, the mean eradication time lengthens and approaches the maximum simulation time of 100 days (color figure online)

For the mean eradication time analysis of the Black Sigatoka Disease in Fig. 11, 500 independent realizations of the model were simulated up to a fixed time horizon of $t_{\max }=100$ days. In each realization, the time to disease eradication was recorded when all infection-related compartments vanished. The mean eradication time was then computed across all realizations, providing a robust measure of eradication dynamics that accounts for both early eradication events and long-term persistence. Figure 11 shows that when the initial host biomass H(0)+R(0) is small, the disease tends to die out relatively quickly. However, as the biomass increases, eradication becomes progressively slower, with the mean eradication time approaching the maximum simulation time $t_{\max }=100$ days. This reflects the biological intuition that larger host populations provide more opportunities for pathogen persistence, making eradication less likely and more delayed.

These results also have implications for post-sanitation monitoring. In particular, the sharp decline in eradication probability and the increase in mean eradication time with host biomass suggest the existence of operational host-density thresholds below which follow-up sanitation and surveillance are most likely to succeed. Thus, maintaining plantations below critical post-intervention host biomass levels may improve the probability of pathogen fade-out and reduce the monitoring period required to confirm eradication.

### Sobol Sensitivity Analysis of Variability of $I^*$

To quantify how parameter uncertainty influences the endemic-infected leaf biomass $I^*$, we perform a global sensitivity analysis using Sobol’s variance-based method (Saltelli et al. 2004; Sobol 2001; Sobol’ 1990). This approach decomposes the variance of the model output into contributions from individual parameters and their interactions, without assuming monotonic relationships. Parameters are sampled over a prescribed range using Sobol sequences, and $I^*$ is computed for each sample to estimate $\textrm{Var}(I^*)$. The first-order Sobol index $(F_i)$ measures the direct contribution of a parameter to output variability, while the total-order index ($T_i$) accounts for both direct effects and all interaction effects.

**Fig. 12:**
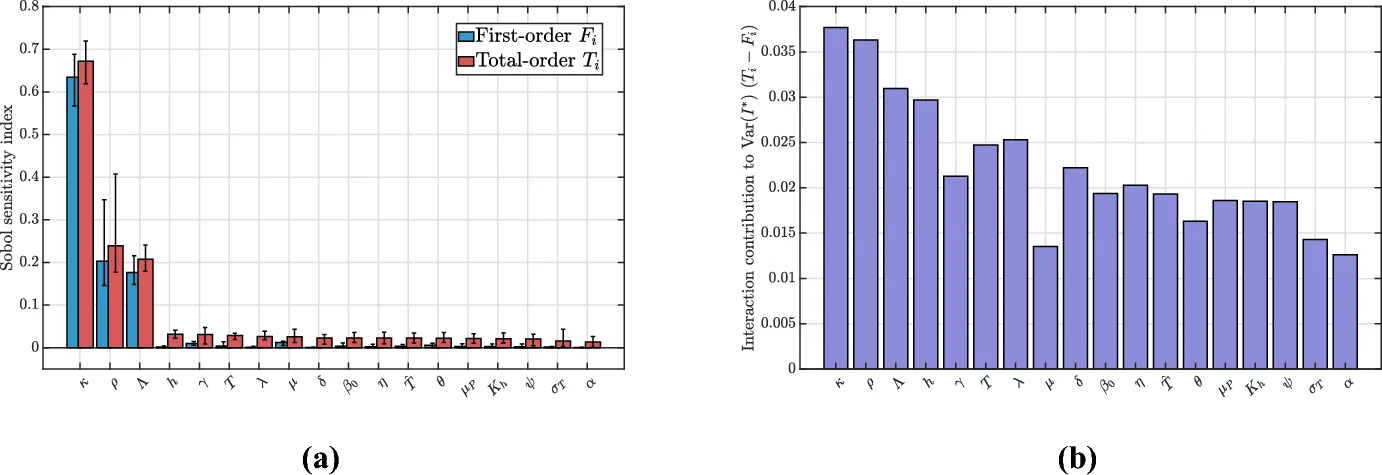
Variance-based global sensitivity analysis of the endemic-infected leaf biomass $I^*$ using Sobol indices. **a** shows the first-order $(S_i)$ and total-order $(S_{T_i})$ sensitivity indices, quantifying the direct and total contributions of each parameter to $\textrm{Var}(I^*)$, respectively. **b** shows the interaction contribution $S_{T_i} - S_i$, highlighting the extent of nonlinear interactions among parameters. Generated using the parameter values stated in Table 2 (color figure online)

Figure 12a shows that the host recruitment parameters κ and Λ, along with (ρ), sanitation, and cultural practices, are the primary drivers of variability in $I^*$. In particular, κ dominates, contributing more than 60% of the variance independently and nearly 70% when interactions are included. Most other parameters exhibit negligible first-order effects, indicating limited individual influence on $I^*$. Figure 12b presents the interaction contributions $T_i-F_i$, showing that the variability of $I^*$ is influenced by distributed nonlinear interactions rather than a single interaction pathway. Although κ, Λ, and ρ remain important, interaction effects are shared across multiple parameters, indicating that the variability of $I^*$ is driven by host recruitment and sanitation processes, with additional contributions from widespread nonlinear interactions. These results suggest that interventions targeting these key parameters are likely to produce robust reductions in endemic infection levels.

## Conclusion

In this study, a deterministic compartmental model incorporating dual transmission pathways (via ascospores and conidia) and mate limitation in sexual reproduction was developed to describe the dynamics of Black Sigatoka disease (BSD) caused by *Pseudocercospora fijiensis*. The model exhibits a backward bifurcation, by which a stable endemic equilibrium coexists with the disease-free equilibrium for a range of parameter values where the basic reproduction number satisfies $\mathcal {R}_0<1$. This bistability provides a theoretical explanation for the observed resilience of BSD. Management strategies that focus on reducing $\mathcal {R}_0$ below 1 may prove insufficient in the backward bifurcation regime, as the system can remain trapped in the endemic state despite a subcritical transmission potential.

Sensitivity analysis of the endemic equilibrium, conducted using normalized forward sensitivity indices, Latin Hypercube Sampling, and Partial Rank Correlation Coefficients, identified the most influential parameters. These results emphasize that effective long-term suppression requires interventions extending beyond $\mathcal {R}_0$ reduction. Priority should be assigned to (1) cultural practices that limit the production of new susceptible leaves during periods of elevated transmission risk, e.g., through optimized planting density, timing, or defoliation strategies, and (2) the development and widespread deployment of genetically resistant or tolerant banana varieties, which directly reduce host susceptibility and therefore weaken both transmission pathways. From a management perspective, these results in Figs. 6, 7, and 12 indicate that interventions aimed at reducing the fraction of newly susceptible leaves (κ), lowering host recruitment (Λ), and strengthening sanitation/cultural practices (ρ) are likely to produce the largest reductions in endemic infected biomass, especially during periods of elevated humidity and near-optimal temperature.

To account for intrinsic stochastic fluctuations inherent in finite populations and discrete infection events, a stochastic model was formulated using the Stochastic Simulation Algorithm (SSA). Extensive numerical realizations demonstrated that stochastic trajectories generally align with deterministic predictions under moderate to high inoculum pressure. However, at low population sizes, stochastic effects introduce substantial variability, including elevated eradication probabilities even when the deterministic model predicts persistence. Sobol variance-based analysis further demonstrates that variability in endemic-infected leaf biomass is primarily driven by a small set of parameters, with additional contributions from nonlinear interactions. These findings highlight the importance of considering demographic stochasticity when evaluating eradication feasibility, particularly in small or recently sanitized plantations. The stochastic analysis demonstrates that deterministic threshold quantities should be interpreted cautiously for finite plantations. While the basic reproduction number correctly predicts invasion in the deterministic limit, stochastic fade-out remains possible when host biomass is sufficiently small. Consequently, successful management depends not only on reducing $\mathcal {R}_0$, but also on decreasing infected biomass below the stochastic persistence threshold.

Beyond identifying resistant cultivars and improving cultural practices as effective management strategies, our results highlight the importance of understanding the reproductive biology of *Pseudocercospora fijiensis*. In particular, monitoring pathogen density and the distribution of compatible mating types may provide valuable information on the likelihood of successful sexual reproduction and long-distance pathogen persistence. The density-dependent mate limitation incorporated into the model demonstrates that Allee effects can substantially influence epidemic dynamics by suppressing sexual reproduction at low pathogen densities while promoting persistence once sufficient infected biomass has accumulated. Consequently, disease management strategies should not focus solely on reducing the basic reproduction number but should also aim to reduce pathogen populations below the critical density required for sustained sexual reproduction. These findings provide new biological insight into the persistence of Black Sigatoka Disease and suggest that incorporating mating dynamics into surveillance programs may improve predictions of epidemic risk and the long-term effectiveness of management interventions.

An important implication of this work is that pathogen persistence depends not only on environmental conditions and host susceptibility but also on the pathogen’s reproductive ecology. The interaction between dual spore transmission pathways and density-dependent mate limitation generates an Allee effect that fundamentally alters epidemic behavior by allowing disease persistence even when the basic reproduction number falls below unity. This finding suggests that future surveillance programs should monitor not only disease incidence but also pathogen density and mating-type distributions, as these quantities may provide early indicators of whether pathogen populations are approaching the critical threshold required for sustained sexual reproduction. Incorporating these biological processes into disease management may improve predictions of epidemic persistence and lead to more effective strategies for the long-term control of Black Sigatoka Disease.

## Data Availability

Data were not used in this study. The codes used for simulations are available on GitHub.
